# Cordidepsine is A Potential New Anti-HIV Depsidone from *Cordia millenii*, Baker

**DOI:** 10.3390/molecules24173202

**Published:** 2019-09-03

**Authors:** Rostanie Dongmo Zeukang, Xavier Siwe-Noundou, Maurice Tagatsing Fotsing, Turibio Tabopda Kuiate, Joseph Tanyi Mbafor, Rui W. M. Krause, Muhammad Iqbal Choudhary, Alex de Théodore Atchadé

**Affiliations:** 1 Department of Organic Chemistry, University of Yaoundé I, Faculty of Science, P.O. Box 812 Yaoundé, Cameroon (R.D.Z.) (M.T.R.) (T.T.K.) (J.T.M.); 2 International Center for Chemical and Biological Sciences, H.E.J Research Institute of Chemistry, University of Karachi, Karachi-75270, Pakistan; 3 Department of Chemistry, Rhodes University, Grahamstown 6140, South Africa

**Keywords:** *Cordia millenii*, isolation, depsidone, cordidepsine, monoglycerol, allotrope sulfur, anti-HIV activity

## Abstract

Chemical investigation of *Cordia millenii,* Baker resulted in the isolation of a new depsidone, cordidepsine (**1**), along with twelve known compounds including cyclooctasulfur (**2**), lup-20(29)-en-3-triacontanoate (**3**), 1-(26-hydroxyhexacosanoyl)glycerol (**4**), glyceryl-1-hexacosanoate (**5**) betulinic acid (**6**), lupenone (**7**), *β*-amyrone (**8**), lupeol (**9**), *β*-amyrin (**10**), allantoin (**11**), 2′-(4-hydroxyphenyl)ethylpropanoate (**12**) and stigmasterol glycoside (**13**). Hemi-synthetic reactions were carried out on two isolated compounds (**5** and **6**) to afford two new derivatives, that is, cordicerol A (**14**) and cordicerol B (**15**), respectively. The chemical structures of all the compounds were established based on analysis and interpretation of spectroscopic data such as electron ionization mass spectrometry (EI–MS), high resolution electrospray ionization mass spectrometry (HR-ESI–MS), fast atom bombardment mass spectrometry (FAB–MS), one dimension and two dimension nuclear magnetic resonance (1D and 2D-NMR) spectral data as well as X-ray crystallography (XRC). Lupeol ester derivatives [Lup-20(29)-en-3-triacontanoate (**3**)], monoglycerol derivatives [1-(26-hydroxyhexacosanoyl)glycerol (**4**) and glyceryl-1 hexacosanoate (**5**)] were isolated for the first time from *Cordia* genus while sulfur allotrope [cyclooctasulfur (**2**)] was isolated for the first time from plant origin. Biological assays cordidepsine (**1**) exhibited significant anti-HIV integrase activity with IC_50_ = 4.65 μM; EtOAc extract of stem barks, EtOAc fraction of roots and leaves were not toxic against 3T3 cells.

## 1. Introduction

Acquired Immune Deficiency Syndrome (AIDS) is one of the major viral infections ravaging the world; around 76.1 million people have been infected since the first report in 1981 [1]. According to the report of the Cameroon Population Based HIV Impact Assessment (CAMPHIA) in 2018, viral prevalence in Cameroon is 3.4% [2] and about 37 million people around the world are currently living with the infection [1]. The absence of vaccines and associated resistance of virus to antiretroviral drugs [1] have contributed to the impairment of most patient’s defense system by opportunistic bacterial infections such as tuberculosis and other bacterial pneumonia infections [3]. 

Bacterial infections are one of the major causes of morbidity and mortality in developing countries [4]. Though antimicrobial drugs have improved the healing of some patients with bacterial infections and reduce mortality, increasing microbial resistance is a major concern [4]. The resistance to treatment may be due to the new interference mechanisms with antimicrobial activity and the emergence of some conditions that weaken the patient’s innate immunity [4]. Since antimicrobial drugs remain necessary for the treatment of many diseases, it is imperative to search for new and efficient ones that may reduce bacterial resistance. The current work identified potential antibacterial agents from *Cordia millenii*.

*Cordia millenii*, Baker is a tree belonging to the Boraginaceae family. It is widely distributed in tropical Africa and is usually planted in West Africa, where various parts are used in traditional medicine [5]. The seed powder, for example, is mixed with palm oil and applied externally to treat ringworm [5]. In Cameroon and other West African countries, decoctions of leaves are applied to expulse worms, to treat coughs, asthma and the common cold. The concoction of its leaves with *Centella asiatica* is used to treat children’s convulsions [6]. A previous phytochemical study of the heartwood of *Cordia millenii* led to the isolation of terpenoids and benzoquinones (Cordichromes A–F) [7]. A general phytochemical screening analysis revealed the presence of phenolic compounds, alkaloids, terpenoids, steroids, glycosides, flavonoids, tannins and saponins [8]. In addition, a number of alkaloid compounds in *C. millenii* are proposed as a basis for developing antimalarial and antibacterial drugs [8]. Moreover, previous studies showed that many species of *Cordia* are used as antiviral drugs. In this instance, the aqueous leaf extract of *Cordia spinescens* had demonstrated potential HIV reverse transcriptase enzyme inhibition with IC_50_ values of 6–8 μg/mL [9]. Several plants of the *Cordia* genus are used for antibacterial, antioxidant, anti-inflammatory, antidiabetic, antitumor purposes and as an analgesic and for wound healing [9]. We report here the isolation and structural identification of fifteen compounds from *C. millenii* and some hemi-synthetic derivatives. The anti-HIV, cytotoxicity and antibacterial activities of crude extract and isolated compounds were investigated to support the traditional use of *C. millenii* for the treatment of infectious diseases.

## 2. Results

### 2.1. Identification of Isolated Compounds

**Figure 1:**
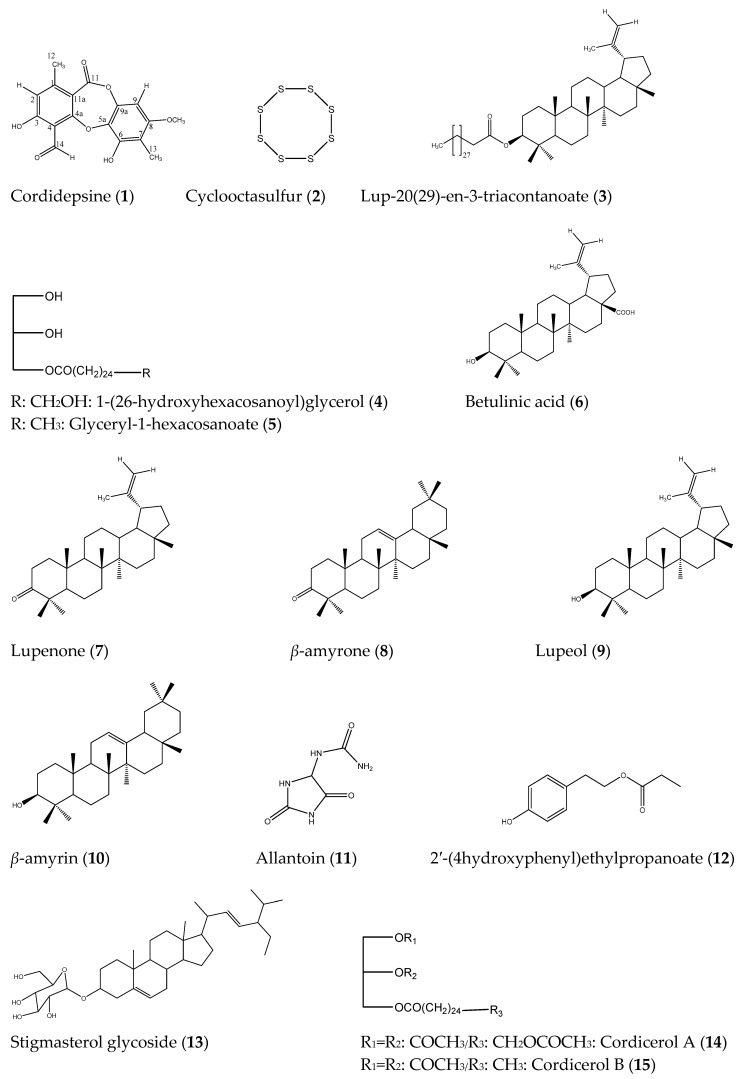
Chemical structures of compounds.

The extracts of *Cordia millenii*, Baker (leaves, stem barks and roots) were submitted to repeated column chromatography to afford one new depsidone (**1**), together with twelve known compounds (Figure 1). These known compounds include: Cyclooctasulfur (**2**), lup-20(29)-en-3-triacontanoate (**3**) [10], lupeol ester (reported for the first time from *Cordia* genus), 1-(26-hydroxyhexacosanoyl)glycerol (**4**) [11], glyceryl-1-hexacosanoate (**5**) [12], which are monoglycerides and this is the first report of monoglycerol from *Cordia* genus. The other known compounds are betulinic acid (**6**) [13], lupenone (**7**) [14], *β*-amyrone (**8**) [15], lupeol (**9**) [16], *β*-amyrin (**10**) [17], allantoin (**11**) [18], 2′-(4-hydroxyphenyl)ethylpropanoate (**12**) [19] and stigmasterol glycoside (**13**) [20]. The chemical structures of the hemi-synthetic derivatives, that is, Cordicerol A (**14**) and Cordicerol B (**15**), are also presented in Figure 1.

**Figure 2:**
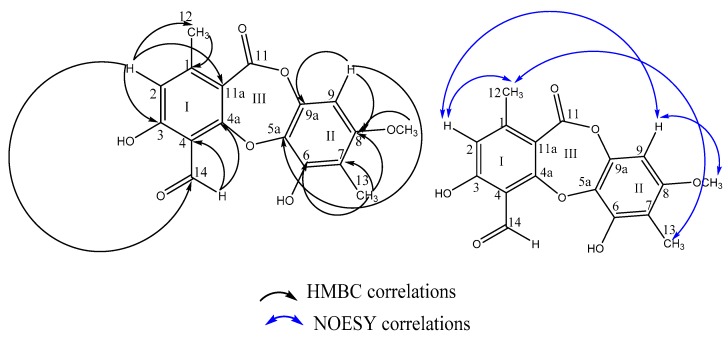
Selected 2D-NMR correlations of Cordidepsine.

Compound **1** was isolated as a white powder from *n*-hexane/EtOAc (6:4, *v*/*v*). Its molecular formula was established as C_17_H_14_O_7_, on the basis of the HR-ESI-MS at *m*/*z* 376.4972 [M + 2Na]^+^ (calcd C_17_H_14_O_7_Na_2_, 376.0535), accounting for 11 double bond equivalents. The ^13^C nuclear magnetic resonance (NMR) spectrum (Table 1) of **1** exhibited the presence of 17 carbon signals, including one methoxy carbon at δ_C_ 56.3 (OCH_3_), two aromatic methyl signals at δ_C_ 21.7 (C-12) and 9.8 (C-13). In addition, we observed in the downfield shield, two aromatic methine signals at δ_C_ 117.4 (C-2) and 107.7 (C-9). This spectrum also revealed the presence of ten aromatic quaternary carbons, of which six oxygenated appeared at δ_C_ 164.0 (C-3), 164.8 (C-4a), 142.5 (C-5a), 161.0 (C-9a), 143.4 (C-6), 154.7 (C-8) and the rest at δ_C_ 152.7 (C-1), 110.9 (C-4), 122.8 (C-7), 111.9 (C-11a). The other signals were attributed to the lactone carbonyl at δ_C_ 166.1 (C-11) and aldehyde function at δ_C_ 193.9 (C-14). These data were similar to those of related depsidones previously isolated from several microorganisms [21,22,23,24] and some species of *Garcinia* genus [25]. The ^1^H NMR spectrum (Table 1) confirmed the presence of two aromatic singlets at δ_H_ 6.85 (1H, *s*, H-2) and δ_H_ 7.08 (1H, *s*, H-9), suggesting two penta-substituted aromatic rings. The ^1^H NMR also displayed two aromatic methyl signals at δ_H_ 2.45 (3H, *s*, H-12), 2.19 (3H, *s*, H-13), one methoxy group signal at δ_H_ 3.85 (3H, *s*, OCH_3_) and the signal of aldehyde proton at δ_H_ 10.43 (1H, *s*, H-14). The HMBC correlations (Figure 2) of H-2 (δ_H_ 6.85) to C-3 (δ_C_ 164.0), C-14 (δ_C_ 193.5), C-11a (δ_C_ 111.9), C-12 (δ_C_ 21.7) suggested that the aldehyde function and the aromatic methyl at δ_C_ 21.7 are located on the same aromatic ring (I). The position of the aldehyde function was also supported by the correlation of H-14 (δ_H_ 10.4) with C-4 (δ_C_ 110.9) and C-4a (δ_C_ 164.8), indicating that the aldehyde function is located at C-4 (δ_C_ 110.9) as evidenced by the above HMBC correlations. In addition, the substituents of the second aromatic ring (II) were identified through HMBC correlations of H-9 (δ_H_ 7.08) to C-5a (δ_C_142.5), C-7 (δ_C_ 122.8), C-8 (δ_C_ 154.7), C-11 (δ_C_ 166.1) and C-13 (δ_C_ 9.8), as well as the HMBC correlations of H-13 to C-6; C-7; and C-8. The assignments of protons to carbon were further confirmed on the HSQC spectrum. However, the aldehyde correlations were shifted to around 30 ppm and the proton at δ_H_ 10.43 (Figure S5, Supplementary materials) due to spectral “folding” of aldehyde signal resulting from a poorly selected ^13^C spectral width applied to reduce the recording time [26]. The required degrees of unsaturation suggested that the aromatic rings (I) and (II) should be linked by an ether and an ester bridge confirming a depsidone skeleton with a seven-membered ring (III) [23]. The proposed structure was reinforced by a NOESY experiment which revealed the correlations (Figure 2) between the methoxy group (δ_H_ 3.85) and H-9 (δ_H_ 7.08), between both methyl groups (δ_H_ 2.19 and δ_H_ 2.45), between aromatic proton at δ_H_ 6.85 (H-2) and methyl protons at δ_H_ 2.45 (H-12); and between both aromatic protons (δ_H_ 6.85 and δ_H_ 7.08). From an analysis of all the above data, the structure of compound **1** was established as 3,6-dihydroxy-8-methoxy-1,7-dimethyl-11-oxo-11*H*-dibenzo[b,e] [1,4]dioxepine-4 carbaldehyde which is a new depsidone to which the trivial name Cordidepsine was assigned. To the best of our knowledge, depsidone metabolite is reported here for the first time in the Boraginaceae family and *Cordia* genus.

**Figure 3:**
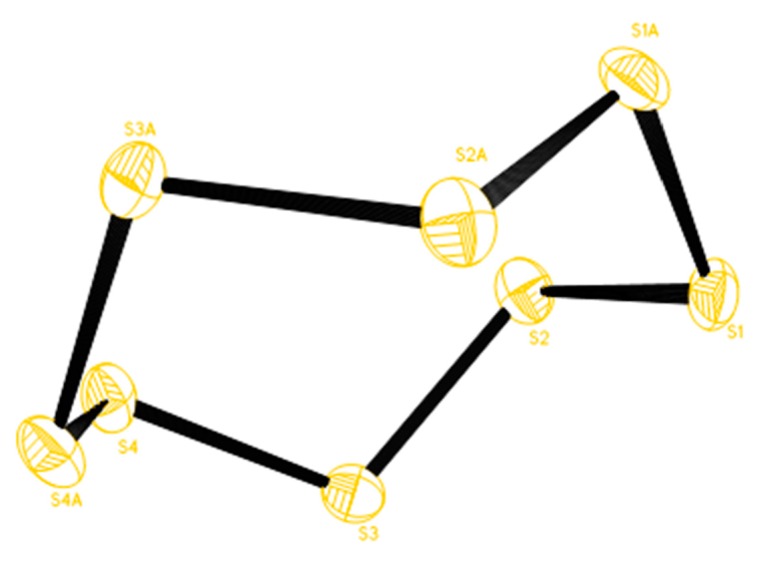
X-ray representation of cyclooctasulfur.

Compound **2** was isolated as a yellow powder from ethyl acetate fraction of roots. Its HR-EI-MS spectrum exhibited a molecular ion peak at *m*/*z* 255.7766, suggesting the molecular formula of S_8_ (calcd 255.7766), which is an allotrope of sulfur. It also presented a difference of *m*/*z* 32 between two consecutive peaks on mass spectral data, suggesting a ^32^S nature of the sulfur. The ^32^S-NMR spectrum was not performed due to the insufficient quantity of isolated sample but its X-ray (Figure 3) was performed and compound **2** was identified as cyclooctasulfur [27]. Cyclooctasulfur was previously reported from a fungal source [28]. To the best of our knowledge, this is the first report of cyclooctasulfur isolated from a plant source. 

### 2.2. Identification of Hemzi-Synthetic Derivatives

**Scheme 1:**
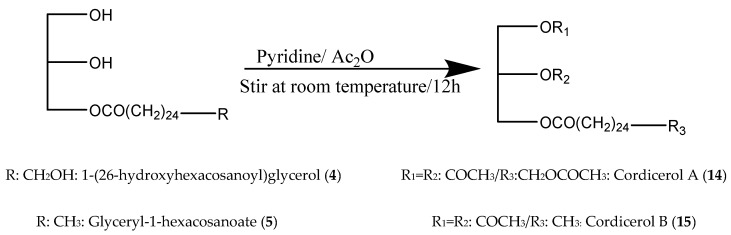
Acetylation reaction of compounds **4** and **5.**

The acetylation reaction (Scheme 1) of isolated compounds **4** and **5** led to the synthesis of new derivatives, that is, cordicerol A (**14**) and cordicerol B (**15**), respectively.

**Figure 4:**
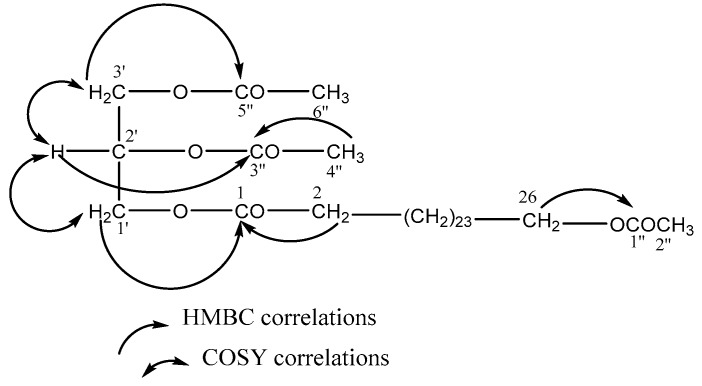
COSY and HMBC correlations for Cordicerol A.

Compound **14** was obtained as a white powder from an acetylation reaction of the hydroxyl groups of compound **4** [1-(26-hydroxyhexacosanoyl)glycerol]. The HR-ESI-MS spectrum (positive-ion mode) exhibited a pseudo-molecular ion [M + H]^+^ peak at *m*/*z* 613.4672, suggesting the molecular formula of C_35_H_64_O_8_ (calcd 613.4679). The ^1^HNMR spectrum (Table 2) revealed signals at δ_H_ 4.03 ppm (2H, *t*, *J* = 6.7 Hz, H-26) attributable to methylene linked to oxygen of ester function; at δ_H_ 2.29 (2H, *t*, H-2) assigned to a methylene at *α* position of carbonyl (C-1) and at δ_H_ 2.02 (3H, *s*, H-2’’); 2.05 (3H, *s*, H-4’’) and 2.06 (3H, *s*, H-6’’), attributable to three methyl groups linked to carbonyl functions, resulted from the acetylation reaction. A broad signal at δ_H_ 1.23 was assigned to the protons of the methylene chain. The ^13^C NMR spectrum (Table 2) displayed signals of four ester groups at δ_C_ 173.3 (C-1), 171.2 (C-1’’), 170.5 (C-5’’) and 170.1 (C-3’’). The structure elucidation was established based on COSY correlations (Figure 4) between methine proton at δ_H_ 5.23 (H-2’) with methylene at δ_H_ 4.13 (H-1’) and 4.28 (H-3’). The structure elucidation was also facilitated by HMBC correlations (Figure 4) between the methine (H-2’) with carbonyl group at δ_C_ 170.1 (C-3’’). HMBC correlations were also observed between methylenes at δ_H_ 4.13 (H-1’), 4.28 (H-3’) and C-1 (δ_C_ 173.3) and C-5’’ (δ_C_ 170.5) respectively, as well as between H-26 (δ_H_ 4.03) and C-1’’ (δ_C_ 171.2). The 2D-NMR correlations (Figure 4) were very important for the determination of the proposed structure. The analysis of 1D and 2D-NMR, HR-ESI-MS data as well as comparison with literature [11] led to the identification of compound **14** as a new derivative of 1-(26-hydroxyhexacosanoyl) glycerol, reported here for the first time. A trivial name—**Cordicerol A**—was given to compound **14.**

**Figure 5:**
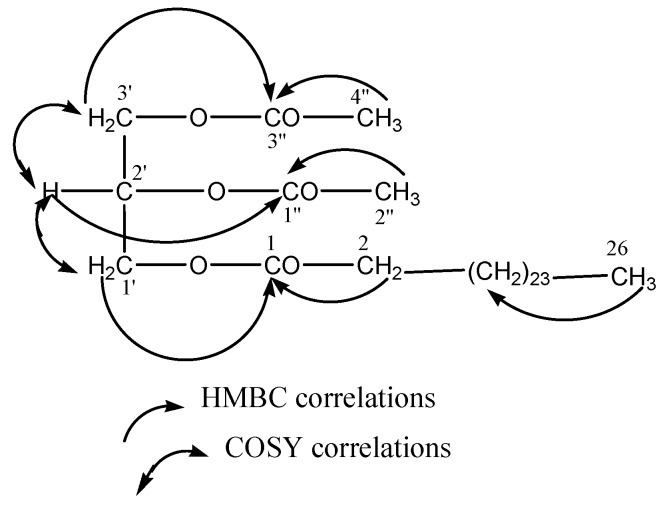
COSY and HMBC correlations for Cordicerol B.

Compound **15** was obtained as a white powder following an acetylation reaction on the hydroxyl groups of compound **5** (glyceryl-1-hexacosanoate). The HR-ESI-MS spectrum (positive-ion mode) of compound **15** exhibited a pseudo-molecular ion [M + Na]^+^ peak at *m*/*z* 577.4448, suggesting the following molecular formula C_33_H_62_O_6_ (calcd 577.4446, C_33_H_62_O_6_Na). The comparison of the spectral data of compounds 14 and 15 (Table 2) shows that the two compounds have the same fatty acyl glycerol skeleton but the ^1^H-NMR spectrum of compound **15** displayed, moreover, a signal of terminal methyl group protons at δ_H_ 0.86 (3H, *t*, *J* = 7 Hz, H-26) and signals of two methyl group linked to carbonyl groups resulted from reaction at δ_H_ 2.06 (3H, *s*, H-2’’) and 2.05 (3H, *s*, H-4’’) instead of three, as observed in compound **14**. The ^13^C NMR spectrum of compound **15** exhibited a signal of three ester groups at δ_C_ 173.3 (C-1), 170.2 (C-1’’) and 170.5 (C-3’’). The presence of a terminal methyl group was confirmed by a signal at δ_C_ 14.1. The analysis of 1D and 2D-NMR (Figure 5), HR-ESI-MS data as well as comparison with literature [12] led to the identification of compound **15** as a new derivative of glyceryl-1-hexacosanoate, reported here for the first time. A trivial name—**Cordicerol B**—was given to compound **15**.

### 2.3. Biological activities 

Many species of *Cordia* are used in traditional medicine for the treatment of various infectious diseases such as malaria, diarrhea, dysentery, stomach pain, fever, blood disorder and syphilis [9]. Due to time constraints, small amounts of samples and availability of assays, antibacterial, cytotoxicity activities of leaves, stem barks and roots crude extracts, fractions and anti-HIV activity of only some isolated compounds were investigated in this study.

#### 2.3.1. Anti-HIV Activity

Cordidepsine (**1**) and allantoin (**11**) were tested in vitro for their inhibitory effect against HIV-1 Integrase. Cordidepsine (**1**) exhibited promising activity with an IC_50_ value of 4.65 μM. Chicoric acid was used as a reference HIV-1 Integrase drug (IC_50_ = 0.33 µM). Allantoin (**11**) also displayed weak activity with an IC_50_ value of 412.94 μM (Table 3). The activity of compound **1** can be justified by the fact that previous biological studies revealed that depsidones are potential antiviral agents [24]. Furthermore, with reference to compound **1**, previous studies showed that an aromatic moiety next to (at least) two adjacent oxygens appears to be a structural element that is essential for activity against HIV-1 integrase [29]. In addition, the activity can also be explained by the presence of aromatic hydroxyl groups which have been reported to be effective inhibitors of integrase [29,30]. Moreover, previous studies showed that many species of *Cordia* are used as antiviral. To this instance, the leaves water extract of *Cordia spinescens* was demonstrated to be a potential inhibitor on HIV reverse transcriptase enzyme with IC_50_ values of 6–8 μg/mL [27].

#### 2.3.2. Antimicrobial Activity 

The different extracts and fractions were tested for their antimicrobial activities (Table 4). EtOAc extracts of roots and stem barks showed good and significant activity against Gram positive bacteria (*Bacillus subtilis* and *Staphylococcus aureus*). These samples were inactive on Gram negative (*Escherichia coli*, *Pseudomonas aeruginosa* and *Salmonella typhi*) whereas EtOAc extract of leaves was not active against all these tested bacteria. This result is in concordance with the previous studies which revealed that species of *Cordia* genus are potential antibacterial agents [9]. The stem bark ethanolic extract of *C. alliodora*, neutral fraction of leaves of *C. cylindrostachya*, leaves and flowers methanolic extracts of *C. boissieri* exhibited antimicrobial activity against Gram positive and negative bacteria [9]. Furthermore, previous studies reported that betulinic acid (**6**), lupenone (**7**), lupeol (**9**) and β-amyrin (**10**) exhibit antibacterial activities [31,32,33] and these reported findings might also explain the observed activities of the extracts.

#### 2.3.3. Cytotoxicity Activity

The percentage cell viability of ethyl acetate extract of stem barks, ethyl acetate fraction of roots and leaves were 99%, 96% and 95% respectively, compared to reference (Cyclohexamide 93%). Based on these findings, all these extracts did not exhibit obvious cytotoxicity activity against 3T3 cell (Human cells) and therefore, were not submitted to further IC_50_ studies. To the best of our knowledge, this is the first report on the toxicity profile of Cordia *millenii*, Baker in the literature.

## 3. Materials and Methods 

### 3.1. General Experimental Procedures

The NMR spectra (^1^H and ^13^C) were recorded on four different Bruker instruments including 400 MHz, 500 MHz, 600 MHz, 800 MHz. Chemical shifts are given in δ (ppm) value relative to TMS as internal standard. Deuterated solvents were used to dissolve the samples for NMR experiments. The HR-ESI-MS spectra were obtained from Bruker Compact QToF and MAXIS II mass spectrometers. EI-MS and FAB-MS data were recorded on a Jeol JMS HX 110 mass spectrometer. Silica gel (230–400 meshes) was used for column chromatography. Thin Layer Chromatography (TLC) and preparative TLC were performed on precoated silica gel plates (60 F_254_, Macherey-Nagel) using various solvent systems as eluent. Spots were visualized using UV light (λ_max_ 254 and 366 nm) and diluted sulphuric acid (10%).

### 3.2. Plant Materials

The plant materials (leaves, stem bark and root) of *Cordia millenii,* Baker were collected from Batoufam, in the West region of Cameroon in May 2015. A voucher specimen (N° 35142/HNC) was deposited in the National Herbarium of Cameroon in Yaoundé.

### 3.3. Extraction and Isolation of Compounds from Cordia millenii Plant Materials

The stem bark powder (1.85 kg) was extracted by maceration at room temperature for 72 h with three different solvents, that is, *n*-hexane, EtOAc and MeOH, successively. The EtOAc extract (35 g) was subjected to column chromatography (CC) over silica gel, eluted with *n*-hexane, EtOAc and MeOH in the increasing order of polarities to afford 20 fractions. Fraction 1 (90 mg) was purified by CC over silica gel with *n*-hexane/CH_2_Cl_2_ (4:6, *v*/*v*) to afford two subfractions (SF_1_ and SF_2_). SF_1_ (44.8 mg) and SF_2_ (35.6 mg) were purified by CC over silica gel and eluted with *n*-hexane/EtOAc (9.2:0.8, *v*/*v*) and (8.5:1.5, *v*/*v*), respectively. SF_1_ gave lupeol (**9**) (22 mg) and *β*-amyrin (**10**) (12 mg). SF_2_ afforded lupenone (**7**) (44 mg) and *β*-amyrone (**8**) (10 mg). Successive CC followed by preparative TLC of fraction 3 (20 mg) using *n*-hexane/EtOAc (6:4, *v*/*v*) afforded cordidepsine (**1**) (2.7 mg). 1-(26-hydroxyhexacosanoyl)glycerol (**4**) (23.6 mg) was obtained by purification of fraction 4 (31.2 mg) in *n*-hexane/EtOAc (1:1, *v*/*v*). Fraction 5 (80 mg) was purified by CC over silica gel and eluted with EtOAc/MeOH (9:1, *v*/*v*)) to give allantoin (**11**) (52 mg).

The roots powder (2.44 kg) was extracted by maceration at room temperature for 72 h with MeOH. The crude extract (185.6 g) was partitioned with *n*-hexane and EtOAc. The EtOAc fraction (50.23 g) was subjected to CC over silica gel eluted with *n*-hexane, EtOAc and MeOH in the increasing order of polarities to afford 4 subfractions. The precipitation of subfraction 1 (40.4 mg) in *n*-hexane led to cyclooctasulfur (**2**) (38.2 mg) after purification. Subfraction 3 (70.5 mg) was purified by CC and eluted with *n*-hexane/EtOAc (8.5:1.5, *v*/*v*) and (7.5:2.5, *v*/*v*) to give betulinic acid (**6**) (11 mg) and glyceryl-1-hexacosanoate (**5**) (20 mg), respectively. 

The powder from leaves (2.45 kg) was extracted by maceration at room temperature for 72 h in MeOH/CH_2_Cl_2_ (1:1, *v*/v). The crude extract (97.87 g) followed the same procedure as for the root extract to yield 4 subfractions (SF). SF_1_ (13 mg) was purified by CC on silica gel with *n*-hexane/EtOAc (8.5:1.5, *v*/*v*) to give lup-20(29)-en-3-triacontanoate (**3**) (9.8 mg). SF_3_ (16.9 mg) was purified by preparative TLC with *n*-hexane/EtOAc (9:1, *v*/*v*) to afford 2′-(4-hydroxyphenyl)ethylpropanoate (**12**) (12 mg). SF_4_ (60 mg) precipitated in EtOAc to afford stigmasterol glycoside (**13**) (35.8 mg). 

### 3.4. Acetylation Reaction of Compounds **4** and **5**

A mixture of 10 mg of compound **4**, 1 ml of acetic anhydride and 1 ml of pyridine was stirred at room temperature for 12 h. After the end of the reaction, a normal workup procedure was followed: 10 mL chloroform and 10 mL distilled water were added to the reaction mixture, the organic layer was separated and dried under reduce pressure. The resulted product was purified by CC with *n*-hexane/EtOAc (9:1, *v*/*v*) to afford compound **14** (6 mg). The same reaction conditions were applied to compound **5** to afford compound **15** (4.8 mg).

### 3.5. Compound Identification

*Cordidepsine* (**1**): white powder (DMSO), HR-ESI-MS *m*/*z* 376.4972 [M + 2Na]^+^ (calcd for C_17_H_14_O_7_Na_2_, 376.0535), ^1^H-NMR, ^13^C-NMR, HMBC and NOESY see Table 1.

*Cordicerol A* (**14**): white powder (CHCl_3_), HR-ESI-MS *m*/*z* 613.4672 [M + H]^+^ (calcd for C_35_H_65_O_8_, 613.4679), ^1^H-NMR, ^13^C-NMR, HMBC and COSY see Table 2.

*Cordicerol B* (**15**): white powder (CHCl_3_), HR-ESI-MS *m*/*z* 577.4448 [M + Na]^+^ (calcd for C_33_H_62_O_6_Na, 577.4446), ^1^H-NMR, ^13^C-NMR, HMBC and COSY see Table 2.

### 3.6. HIV-1 Integrase Strand Transfer Reaction Assay

The HIV-1 subtype C integrase (CIN) strand transfer inhibition assay was adapted from previously described method [34]. 20 nM double-stranded biotinylated donor DNA (5′-5Biotin TEG/ACCCTTTTAGTCAGTGTGGAAAATCTCTAGCA-3′ annealed to 5′ACTGCTAGAGATTTTCCACACTGACTAAAAG-3′) was immobilized in wells of streptavidin coated 96-well microtiter plates (R&D Systems, USA). Following incubation at room temperature for 40 min and a stringent wash step, 5 μg/mL purified recombinant HIV-1 CIN in buffer 1 (50 mm NaCl, 25 mM Hepes, 25 mM MnCl_2_, 5 mM β-mercaptoethanol, 50 μg/mL BSA, pH 7.5) was added to individual wells. Test samples and chicoric acid were added to individual wells to a final concentration of 20 μM (pure compounds and chicoric acid) and 20 µg/mL (extracts). Recombinant HIV-1 subtype C IN was assembled onto the preprocessed donor DNA through incubation for 45 min at room temperature. Strand transfer reaction was initiated through the addition of 10 nM (final concentration) double-stranded FITC-labelled target DNA (5′-TGACCAAGGGCTAATTCACT/36-FAM/−3′annealed to 5′- AGTGAATTAGCCCTTGGTCA−/36-FAM/−3′) in integrase buffer 2 (same as buffer 1, except 25 mm MnCl_2_ replaced with 2.5 mm MgCl_2_). After an incubation period of 60 min at 37 °C, the plates were washed using PBS containing 0.05% Tween 20 and 0.01% BSA, followed by the addition of peroxidase-conjugated sheep anti-FITC antibody (ThermoScientific, USA), diluted 1:1000 in the same PBS buffer. Finally, the plates were washed and peroxidase substrate (Sure Blue Reserve^TM^, KPL, USA) was added to allow for detection at 620 nm using a Synergy MX (BioTek®) plate reader. Absorbance values were converted to percentage enzyme activity relative to the readings obtained from control wells (enzyme without inhibitor).

### 3.7. Antibacterial Activity

This assay was used to screen the antibacterial activity of the extracts and it was evaluated using the microplate alamar blue method [35,36]. Organisms, grown in Mueller Hinton medium (Oxoid Limited, UK), were inoculated in Mueller Hinton Broth (MHB) (Oxoid Limited, UK) and were incubated overnight at 37 °C. Fully grown turbid bacterial cultures were then diluted to adjust with 0.5 McFarland Turbidity Index (equivalent to 1.5 × 10^8^ CFU/mL). Stock solutions (60 mg/mL) of different extracts were prepared in DMSO and 10 μL each of these stock solutions were placed in wells of flat bottom, polystyrene, sterile 96-wells micro titer plate except the positive control wells (media + bacteria). This gave 3000 μg/mL concentrations of extracts in the final 200 μL solution. Finally, bacterial suspension (3 × 10^6^ CFU/mL) was added in each well. Plates were sealed with parafilm and incubated at 37 °C for 18–24 h. Next day, 20 μL of 0.02% resazurin sodium salt dye (Chem-Impex-Int’L Inc.) was added to each well and was incubated in a shaking incubator at 80 rpm and 37 °C for 2–3 h. The color change from blue to reddish pink indicated the growth of bacteria. For quantitative analysis, plates were read at 570 nm and 600 nm in a Multiskan™ GO microplate spectrophotometer, (ThermoScientific, USA). The % inhibition of bacterial growth was calculated using the formula:

% inhibition = 100 − (% difference in the reduction between treated and positive control bacteria)

### 3.8. Cytotoxicity Activity

It was evaluated in 96-well flat-bottomed micro plates by using the standard MTT (3-[4, 5-dimethylthiazole-2-yl]-2, 5-diphenyl-tetrazolium bromide) colorimetric assay [37]. For this purpose, 3T3 (mouse fibroblast) cells were cultured in Dulbecco’s Modified Eagle Medium, supplemented with 5% of fetal bovine serum (FBS), 100 IU/mL of penicillin and 100 µg/mL of streptomycin in 75 cm^2^ flasks and kept in 5% CO_2_ incubator at 37 °C. Exponentially growing cells were harvested, counted with a hemocytometer and diluted with a particular medium. Cell culture with the concentration of 5 × 10^4^ cells/mL was prepared and introduced (100 µL/well) into 96-well plates. After overnight incubation, medium was removed and 200 µL of fresh medium was added with different concentration of compounds (1–30µM). After 48h, 200 µL MTT (0.5 mg/mL) was added to each well and incubated further for 4h. Subsequently, 100 µL of DMSO was added to each well. The extent of MTT reduction to formazan within cells was calculated by measuring the absorbance at 540 nm, using a micro plate reader (Spectra Max plus, Molecular Devices, CA, USA). The cytotoxicity was recorded as concentration causing 50% growth inhibition (IC_50_) for 3T3 cells. The percentage inhibition was calculated by using the following formula:

% inhibition = 100 − ((mean of O.D of test compound – mean of O.D of negative control)/(mean of O.D of positive control – mean of O.D of negative control)*100).

### 3.9. X-ray Diffraction Studies

Crystals of compound **2** were grown by slow evaporation of methanol/chloroform solution at 293K. The crystal used for X-ray measurement was lamellar, with dimensions of 0.16 × 0.07 × 0.06 mm. Cyclic octaatomic sulfur, S_8,_ M_x_ = 256.48g.mol^−1^ crystallized in the orthorhombic system, space group Fddd (Z = 16). The unit cell parameters were as follow: a = 10.4709 (7) Å, b = 12.8709(8) Å and c = 24.484(2) Å with a cell volume of 3299.7(4) Å^3^. The calculated density equal to 2.065 mg·m^−3^. The linear absorption coefficient was µ = 19.279 mm^−1^ for the λ (M_0_K_α_) radiation (λ = 1.54178 Å). The diffracted intensities were collected with an ENRAF NONIUS Kappa CCD diffractometer. The structure was solved by Direct Methods (SHELXS 97) and refined by Full-matrix least-squares on F^2^ [38]. All sulfur atoms were refined anisotropically. The final reliability factors were R_1_ = 0.0178, *w*R_2_ = 0.0449 [I > 2σ(I)] and the goodness of fit on F^2^ was equal to 1.038. The maximum and minimum transmission were 0.3909 and 0.1484, respectively.

## 4. Conclusions

Column chromatography of stem bark, roots and leaves of *C. millenii* led to the isolation and identification of cordidepsine (**1**), a new derivative of depsidone, cyclooctasulfur (**2**), isolated from plant source and eleven known compounds—lup-20(29)-en-3-triacontanoate (**3**), 1-(26-hydroxyhexacosanoyl)glycerol (**4**), glyceryl-1-hexacosanoate (**5**), betulinic acid (**6**), lupenone (**7**), *β*-amyrone (**8**), lupeol (**9**), *β*-amyrin (**10**), allantoin (**11**), 2′-(4-hydroxyphenyl)ethylpropanoate (**12**) and stigmasterol glycoside (**13**). Two derivatives of monoglycerol were hemi-synthesized through acetylation reactions to afford cordicerol A (**14**) and cordicerol B (**15**). In addition, antibacterial and cytotoxicity as well as anti-HIV-integrase activities in vitro of some samples were evaluated. Cordidepsine (**1**) exhibited interesting in vitro anti-HIV-integrase activity with an IC_50_ value of 4.65 μM. The crude extracts of different parts of *C. millenii* were not toxic against 3T3 cell (human cells) while leaves samples were inactive against all tested bacteria.

## Figures and Tables

**Table 1:** Nuclear magnetic resonance (NMR) spectroscopic data (600 MHz for ^1^H and 150 MHz for ^13^C, in DMSO) of compound **1** (δ in ppm).

Position	δ (^1^H) (*mult*)	δ (^13^C)	HMBC	NOESY
1	-	152.7	-	-
2	6.85 (1H, *s*)	117.4	C-3, C-11a, C-12, C-14	H-9
3	-	164.0	-	-
4	-	110.9	-	-
4a	-	164.8	-	-
5a	-	142.5	-	-
6	-	143.4	-	-
7	-	122.8	-	-
8	-	154.7	-	-
9	7.08 (1H, *s*)	107.7	C-5a, C-7, C-8, C-11, C-13	H-2, OCH_3_
9a	-	161.0	-	-
11	-	166.1	-	-
11a	-	111.9	-	-
12	2.45 (3H, *s*)	21.7	C-1, C-2, C-4a, C-11a	H-13
13	2.19 (3H, *s*)	9.8	C-6, C-7, C-8	H-12
14	10.43 (1H, *s*)	193.9	C-4, C-4a	-
OCH_3_	3.83 (3H, *s*)	56.3	C-8	H-9

**Table 2:** NMR spectroscopic data (500 MHz for ^1^H and 125 and 200 MHz for ^13^C, in CDCl_3_) of compounds **14** and **15** (δ in ppm and *J* in Hz).

14					15			
Position	δ (^1^H) (*mult*, J (Hz)	δ(^13^C)	HMBC	COSY	δ (^1^H) (*mult*, J (Hz)	δ(^13^C)	HMBC	COSY
1	-	173.3	-	-	-	173.3	-	-
2	2.29 (2H, *t*, J = 7.5)	34.0	C-1	-	2.29 (2H, *t*, J = 7.5)	34.0	C-1	-
3-25	1.23–1.60 [(2H)_n_, *m*]	28.5–29.7	-	-	1.23–1.61 [(2H)_n_, *m*]	24.8-29.6	C-26	H-26
26	4.03 (2H, *t*, J = 6.7)	64.6	C-1’’	-	0.86 (3H, *t*, J = 7.0)	14.1	-	H-25
1’	4.13 (2H, *m*)	62.2	C-1	H-2’	4.27 (2H, *m*)	61.9	C-1	H-2’
2’	5.23 (1H, *m*)	69.1	C-3’’	H-1’, H-3’	5.23 (1H, *m*)	69.1	C-1’’	H-1’, H-3’
3’	4.28 (2H, *m*)	61.9	C-5’’	H-2’	4.13 (2H, *m*)	62.3	C-3’’	H-2’
1’’	-	171.2	-	-	-	170.0	-	-
2’’	2.02 (3H, *s*)	20.6	-	-	2.06 (3H, *s*)	21.0	C-1’’	-
3’’	-	170.1	-	-	-	170.5	-	-
4’’	2.05 (3H, *s*)	20.8	C-3’’	-	2.05 (3H, *s*)	20.8	C-3’’	-
5’’	-	170.5	-	-	-	-	-	-
6’’	2.06 (3H, *s*)	21.0	-	-	-	-	-	-

**Table 3:** Anti-HIV-1 integrase activity (IC_50_ in μM) of isolated compounds **1** and **11.**

Compounds	Name	IC_50_
**1**	Cordidepsine	~ 4.65
**11**	Allantoin	~ 412.94
Reference	Chicoric acid	0.33

IC_50_: 50% inhibitory concentration, that is, the concentration of extract/compound that inhibits by 50% the activities of the enzyme .

**Table 4:** In vitro antibacterial activity of *Cordia millenii.*

	Code	Microorganisms Tested ^a^				
		*E. coli*	*B. subtilis*	*S. aureus*	*P. aeruginosa*	*S. typhi*
EtOAc fraction of roots	RCM-A	NI	49.45	NI	NI	NI
EtOAc extract of stem barks	TCM-H	NI	NI	65.2	NI	NI
EtOAc fraction of leaves	FCM	NI	NI	NI	NI	NI
Reference	Tetracycline	-	92.00	89.00	-	-

^a^ The data are represented as percentage (%) of inhibition; NI: No Inhibition.

## Supplementary Materials

The spectra of compounds (**1**, **14** and **15**) and crystal data of compound **2** are available online at https://www.mdpi.com/1420-3049/24/17/3202/s1. Figure S1–S22: MS and NMR spectral data of compounds **1**, **14** and **15**, Table S1–S4: X-ray crystallography data of compound 2.

## Abbreviations

AC_2_O, Acetic anhydride; CC, Column Chromatography, CDCl_3_, Chloroform-deuterated; CH_2_Cl_2_, Dichloromethane; COSY, Correlation Spectroscopy; EI, Electronic Impact; HR-ESI, High Resolution Electrospray Ionization; DEPT, Distortionless Enhancement by Polarization Transfert, DMSO, Dimethyl sulfoxide; EtOAc, Ethyl Acetate; FAB, Fast Atom Bombardment; HMBC, Heteronuclear Multiple Bond Correlation; HNC, Herbier National du Cameroun (i.e. National herbarium of Cameroon); HR, High Resolution; HSQC, Heteronuclear single Quantum Correlation; MeOH, Methanol; MS, Mass Spectroscopy; NOESY, Nuclear Overhauser Effect Spectroscopy; TLC, Thin layer Chromatography; 1D-and 2D-NMR, one dimension and two dimensions nuclear magnetic resonance; v/v, volume by volume.
