# Supplementary material for: Cordidepsine is A Potential New Anti-HIV Depsidone from Cordia millenii, Baker
Source: Molecules. 2019 Sep 3;24(17):3202. doi: 10.3390/molecules24173202 (PMC6749241; doi:10.3390/molecules24173202)
Supplement: Supplementary file 1 [file molecules-24-03202-s001.pdf]

Article

Cordidepsine is a potential new anti-HIV depsidone from *Cordia millenii*, Baker.

Rostanie Dongmo Zeukang [1,2], Xavier Siwe-Noundou [3*], Maurice Tagatsing Fotsing [1], Turibio Tabopda Kuiate [1], Joseph Tanyi Mbafor [1], Rui W. M. Krause[3], Muhammad Iqbal Choudhary [2] and Alex de Théodore Atchadé [1*]

[1] Department of Organic Chemistry, University of Yaoundé I, Faculty of Science, 812 Yaoundé, Cameroon; zeukangrostanie@yahoo.com (R.D.Z.), alexiode@yahoo.fr (A.T.A.), ttabopda@yahoo.fr (T.T.K), ftagat@yahoo.com (M.T.F.), mbaforjt53@gmail.com (J.T.M.)

[2] International Center for Chemical and Biological Sciences, H.E.J Research Institute of Chemistry, University of Karachi, Karachi-75270, Pakistan; iqbal.choudhary@iccs.edu (M.I.C.)

[3] Department of Chemistry, Rhodes University, Grahamstown 6140, South Africa; xavsiw@gmail.com / x.siwenoundou@ru.ac.za (X.S.N.), r.krause@ru.ac.za (R.W.M.K.)

* Correspondence: xavsiw@gmail.com / x.siwenoundou@ru.ac.za (X.S.N.); alexiode@yahoo.fr (A.T.A.)

Received: date; Accepted: date; Published: date

Abstract: Chemical investigation of *Cordia millenii,* Baker resulted in the isolation of a new depsidone, cordidepsine (**1**), along with twelve known compounds including cyclooctasulfur (**2**), lup-20(29)-en-3-triacontanoate **(3)**, 1-(26-hydroxyhexacosanoyl)glycerol(**4)**, glyceryl-1-hexacosanoate (**5**) betulinic acid (**6**), lupenone (**7**), *β*-amyrone (**8**), lupeol (**9**), *β*-amyrin (**10**), allantoin (**11**), 2′-(4-hydroxyphenyl)ethylpropanoate (**12**) and stigmasterol glycoside (**13**). Hemi-synthetic reactions were carried out on two isolated compounds, compounds **5** and **6** to afford two new derivatives i.e. cordicerol A (**14**) and cordicerol B (**15**) respectively. The chemical structures of all the compounds were established based on analysis and interpretation of spectroscopic characterization such as EI–MS, ESI–MS, FAB–MS, 1D and 2D-NMR spectral data as well as X-ray crystallography. Lupeol ester derivatives [Lup-20(29)-en-3-triacontanoate (**3**)], monoglycerol derivatives [1-(26-hydroxyhexacosanoyl)glycerol (**4**) and glyceryl-1 hexacosanoate (**5**)] were isolated for the first time from *Cordia* genus while sulfur allotrope [cyclooctasulfur (**2**)] was isolated for the first time from plant origin. Biological assays such as anti-HIV, cytotoxicity and antibacterial activities were evaluated. Cordidepsine (**1**) exhibited significant anti-HIV integrase activity with IC_{50} ~ 4.65 μM; EtOAc extract of stem barks, EtOAc fraction of roots and leaves were not toxic against 3T3 cell.

Keywords: *Cordia millenii*; Isolation; Depsidone; Cordidepsine; Monoglycerol; Allotrope sulfur; Anti-HIV activity.

Compound 1 (Cordidepsine)

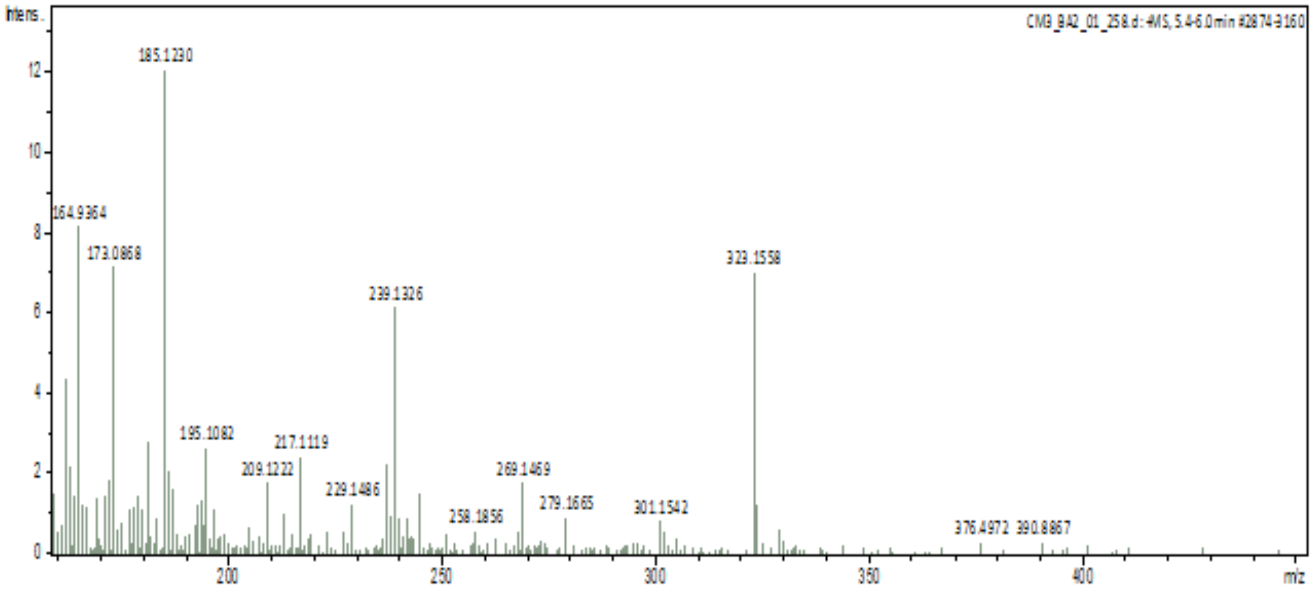

Figure S1. HR-ESI-MS of Cordidepsine (**1**)

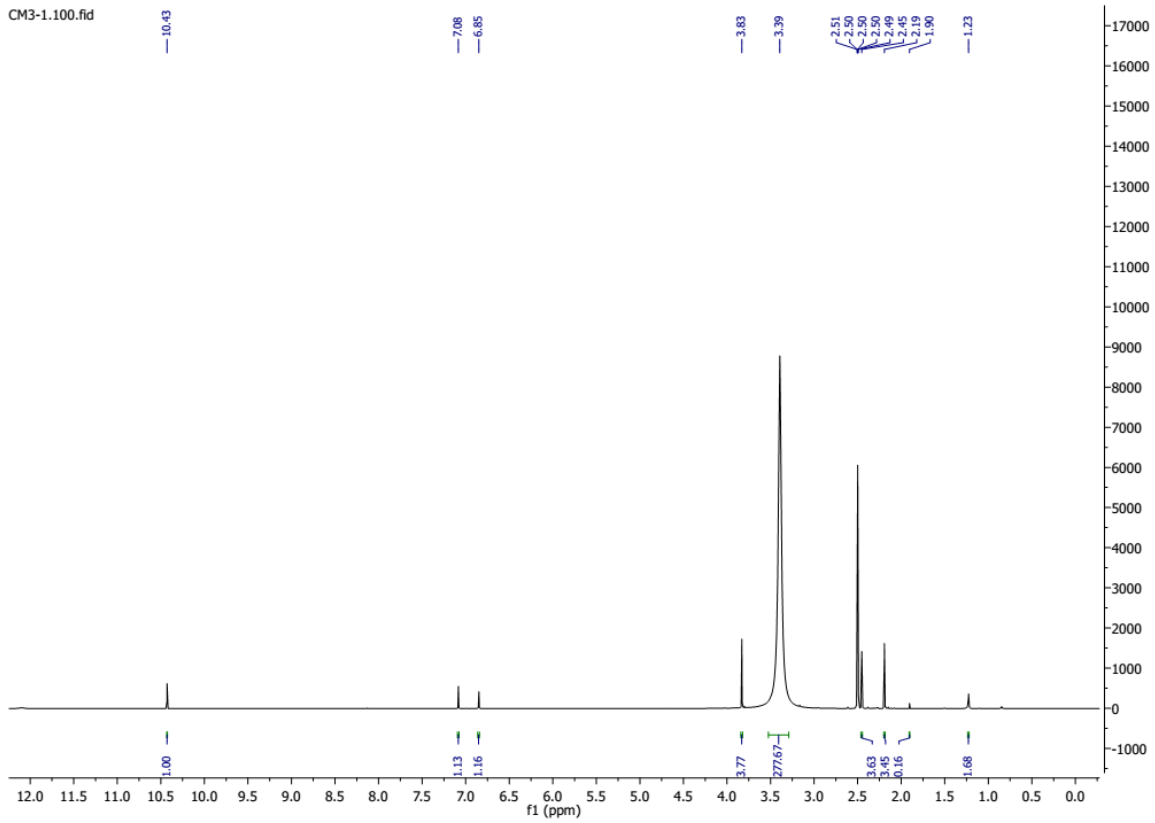

Figure S2. ^{1}H NMR (600 MHz, DMSO) spectrum of Cordidepsine (**1**)

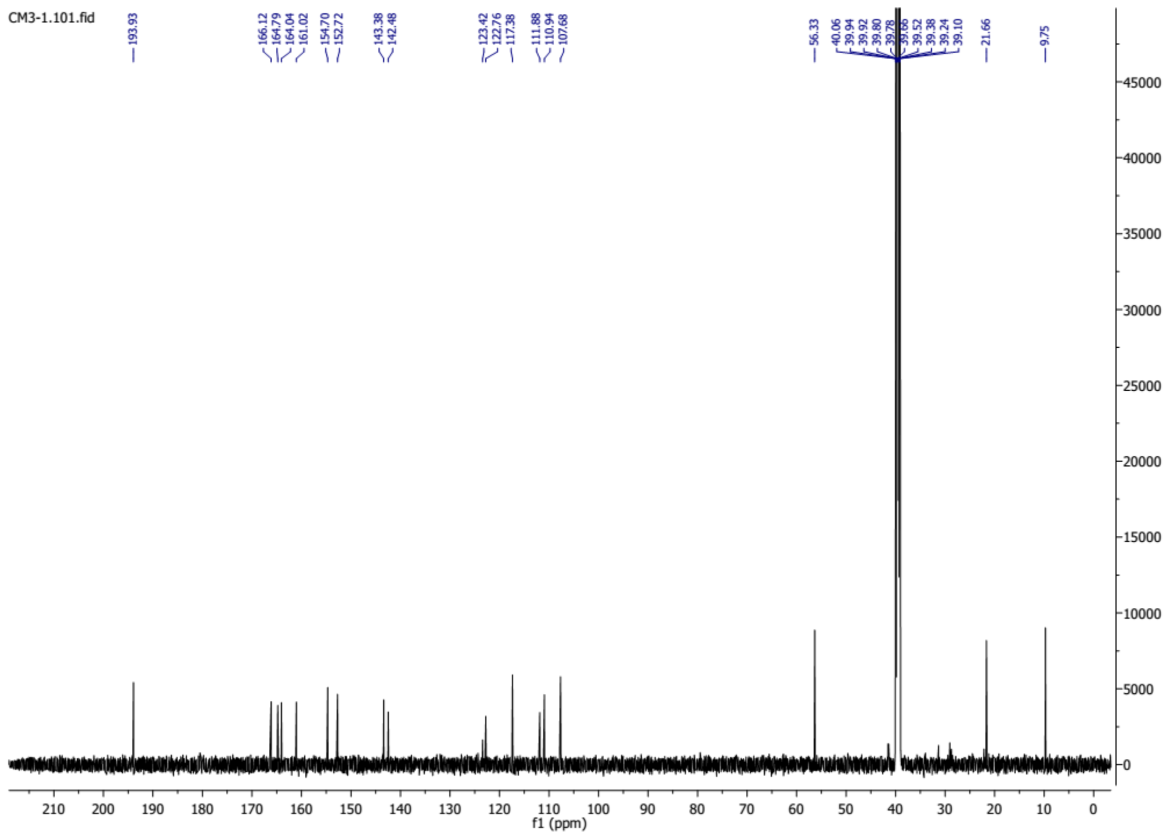

Figure S3. ^{13}C NMR (150 MHz, DMSO) spectrum of Cordidepsine (**1**)

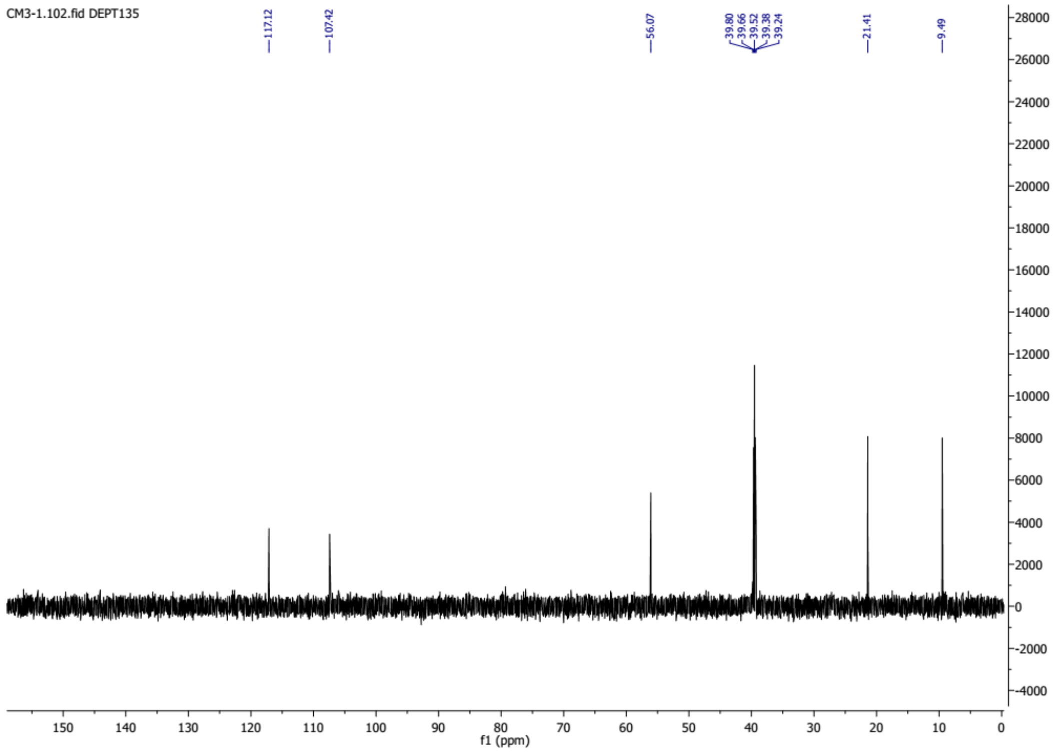

Figure S4. DEPT 135 (150 MHz, DMSO) spectrum of Cordidepsine (**1**)

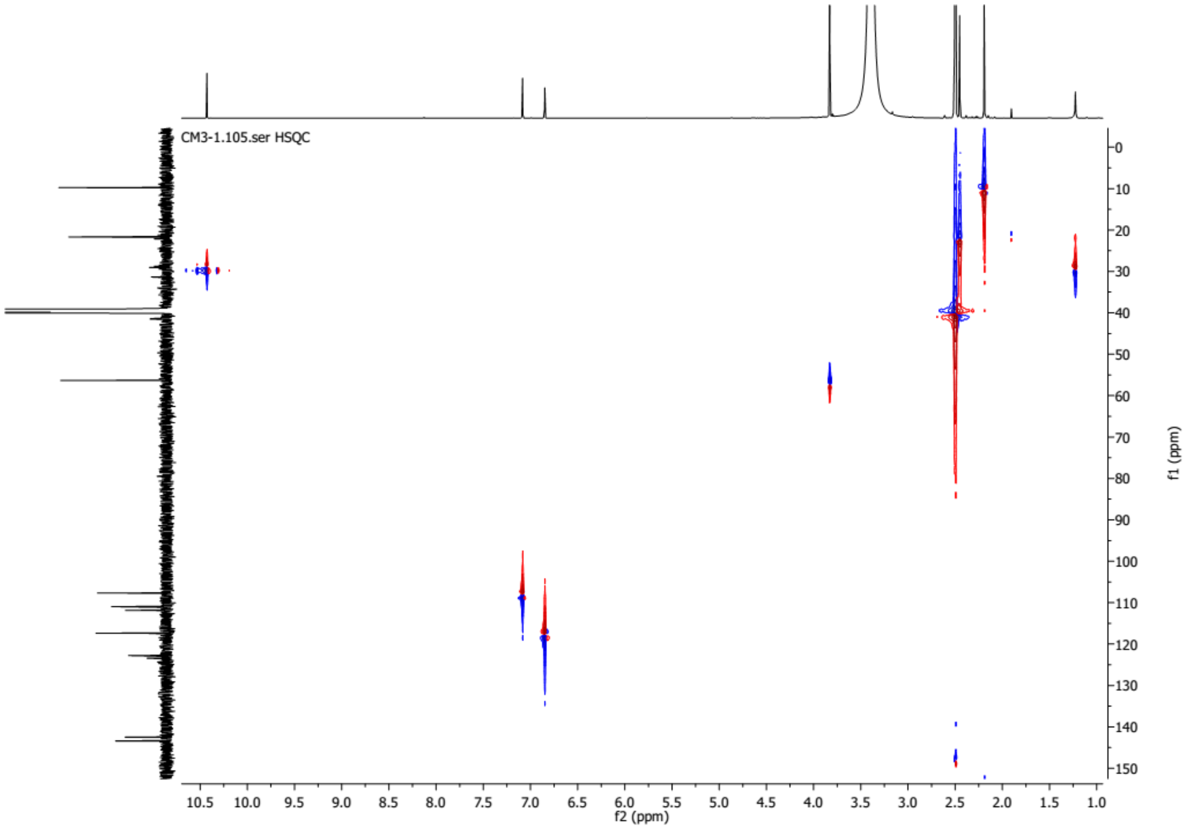

Figure S5. HSQC (^{1}H: 600 MHz, ^{13}C : 150 MHz, DMSO) spectrum of Cordidepsine (**1**)

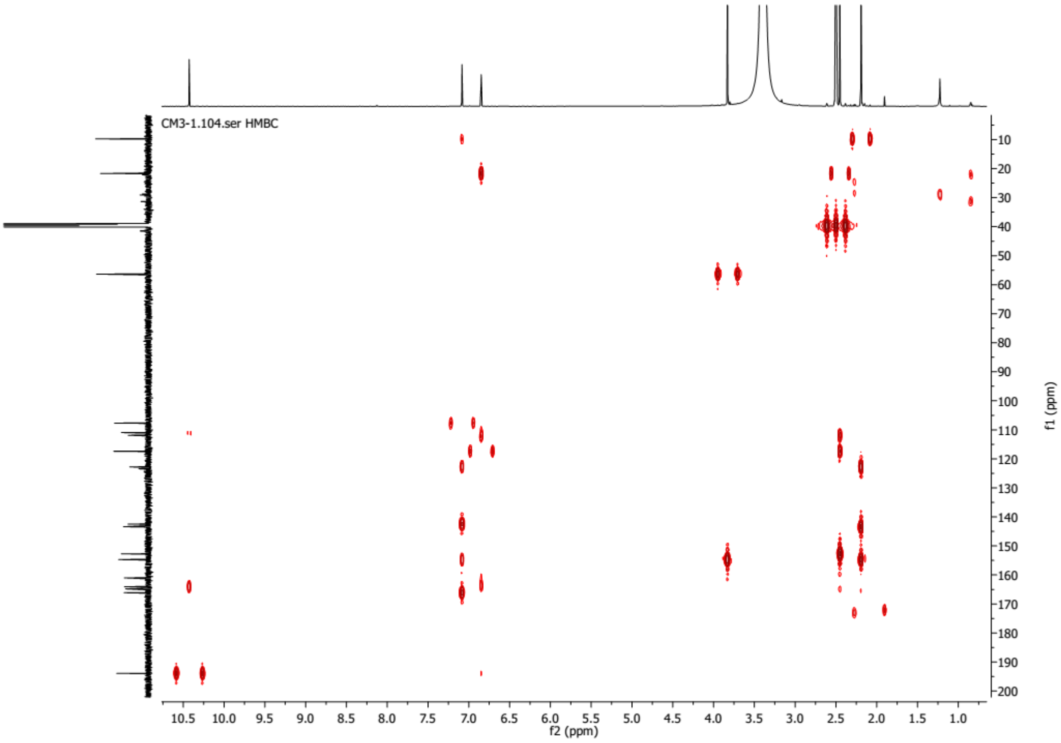

Figure S6. HMBC (^{1}H: 600 MHz, ^{13}C:150 MHz, DMSO) spectrum of Cordidepsine (**1**)

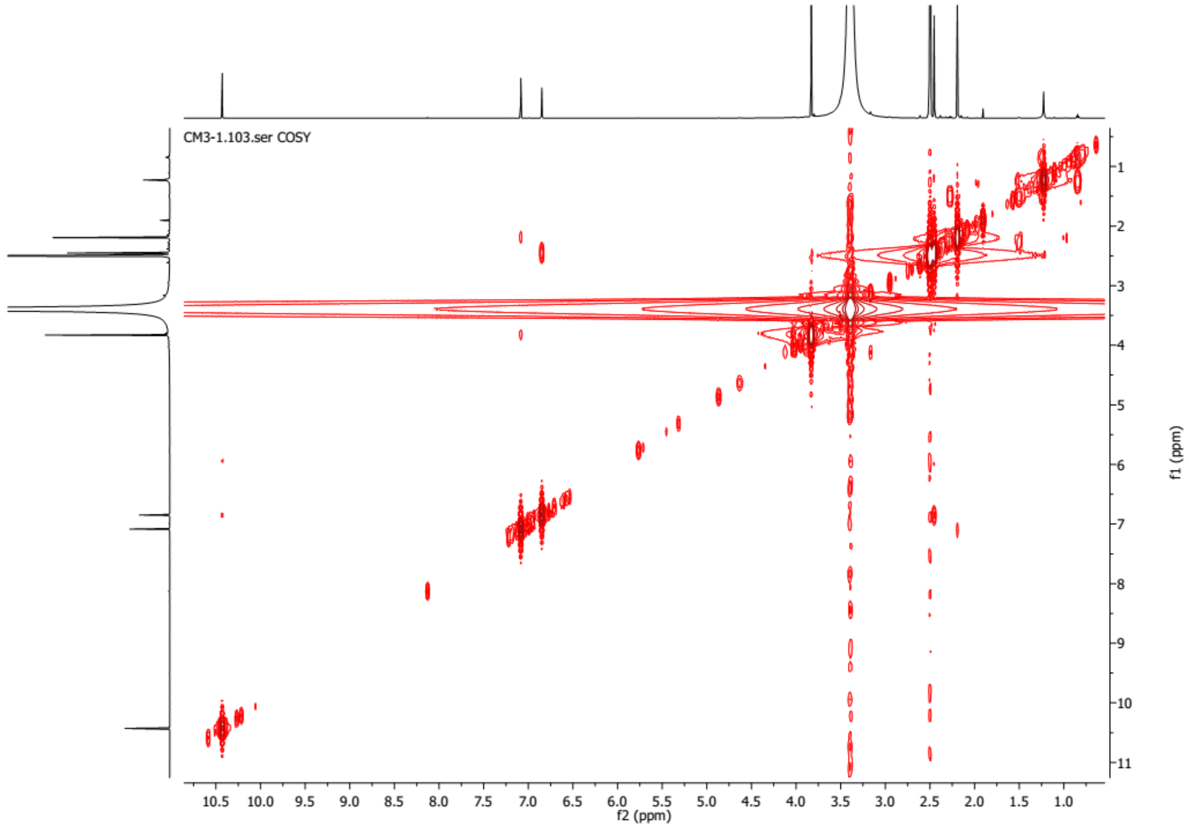

Figure S7. COSY (600 MHz, DMSO) spectrum of Cordidepsine (**1**)

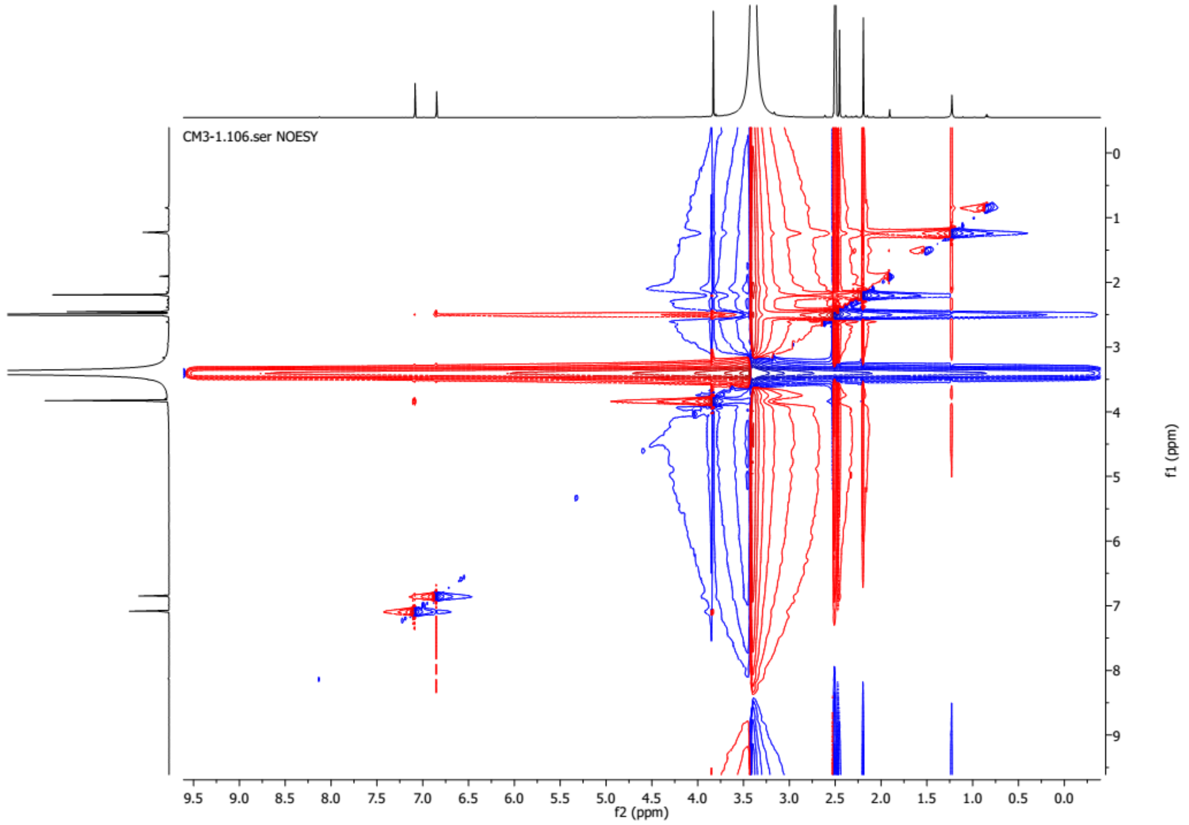

Figure S8. NOESY (600 MHz, DMSO) spectrum of Cordidepsine (**1**)

Compound 14 (Cordicerol A)

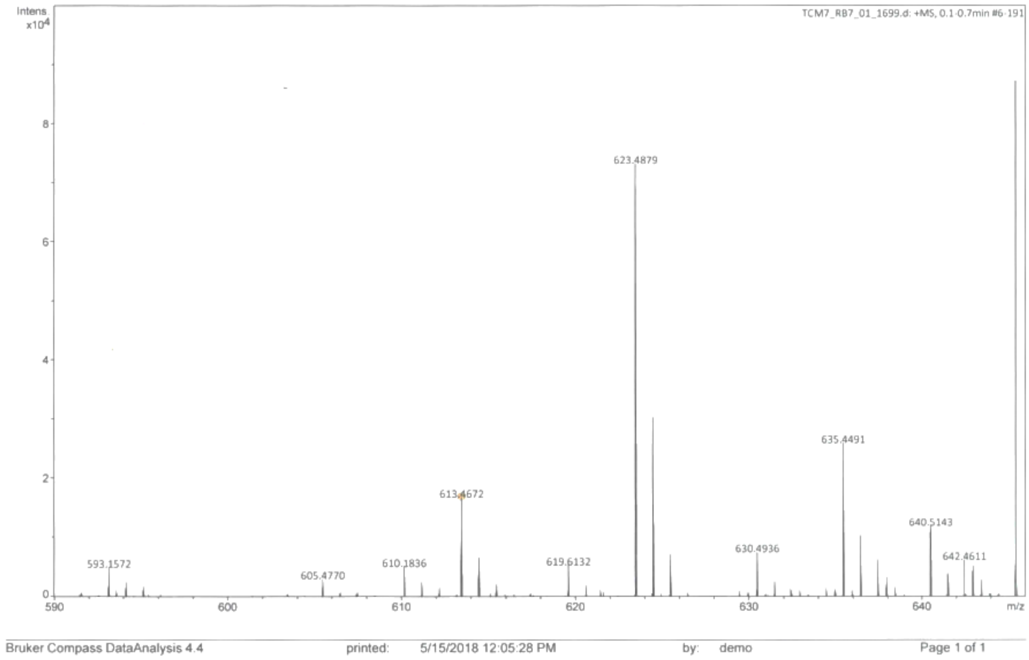

Figure S9. HR-ESI-MS of Cordicerol A (**14**)

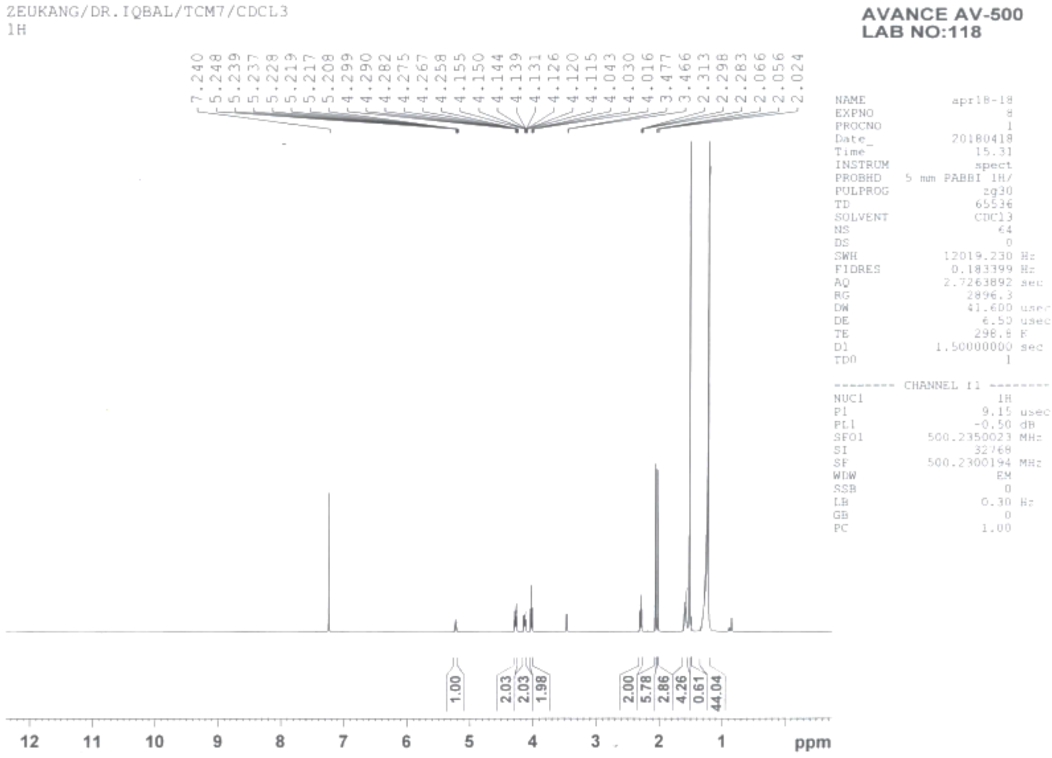

Figure S10. ¹H NMR (500 MHz, $CDCl_3$) spectrum of Cordicerol A (**14**)

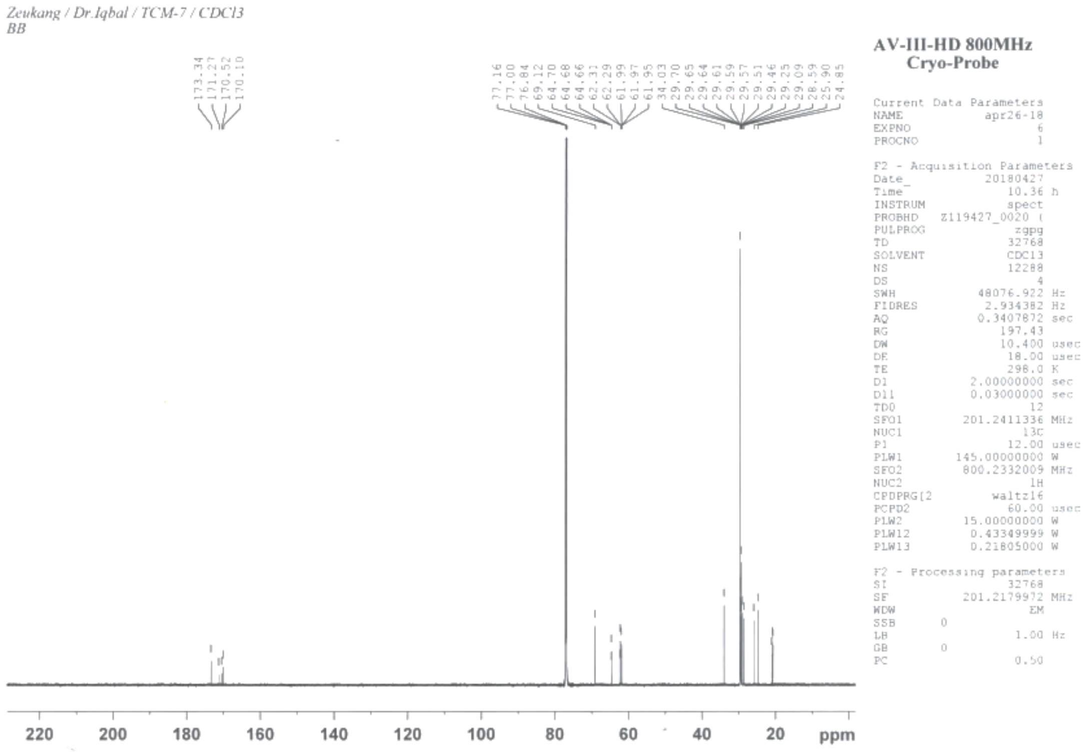

Figure S11. ^{13}C NMR (200 MHz, $CDCl_3$) spectrum of Cordicerol A (**14**)

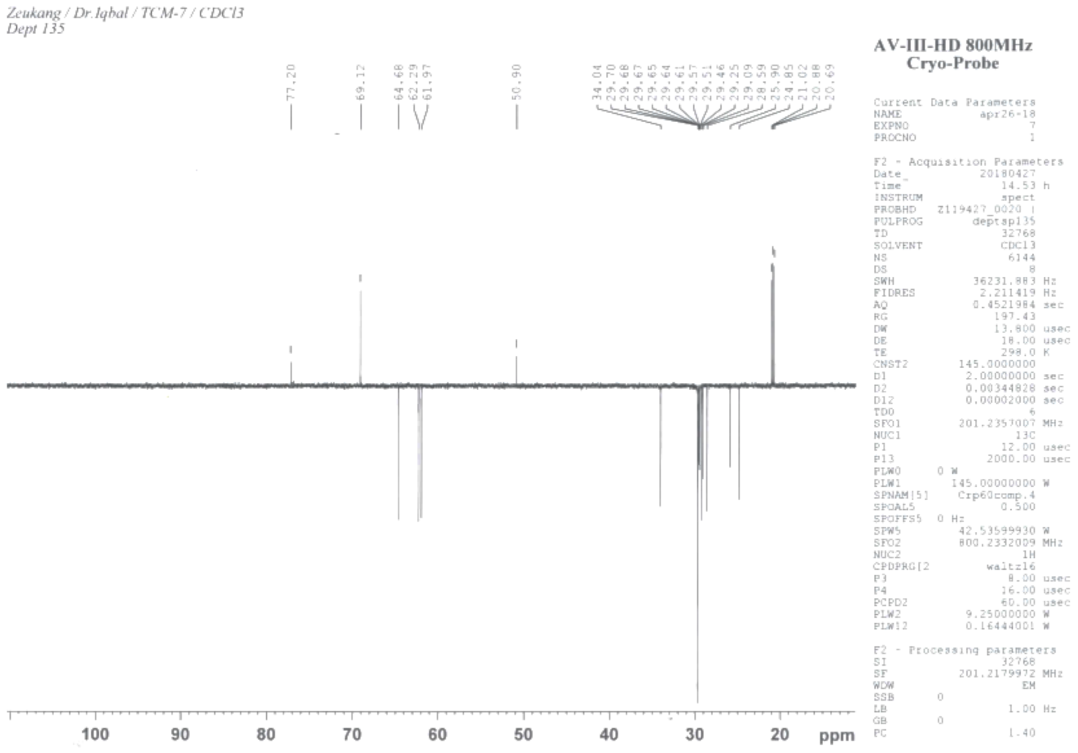

Figure S12. DEPT 135 (200 MHz, $CDCl_3$) spectrum of Cordicerol A (**14**)

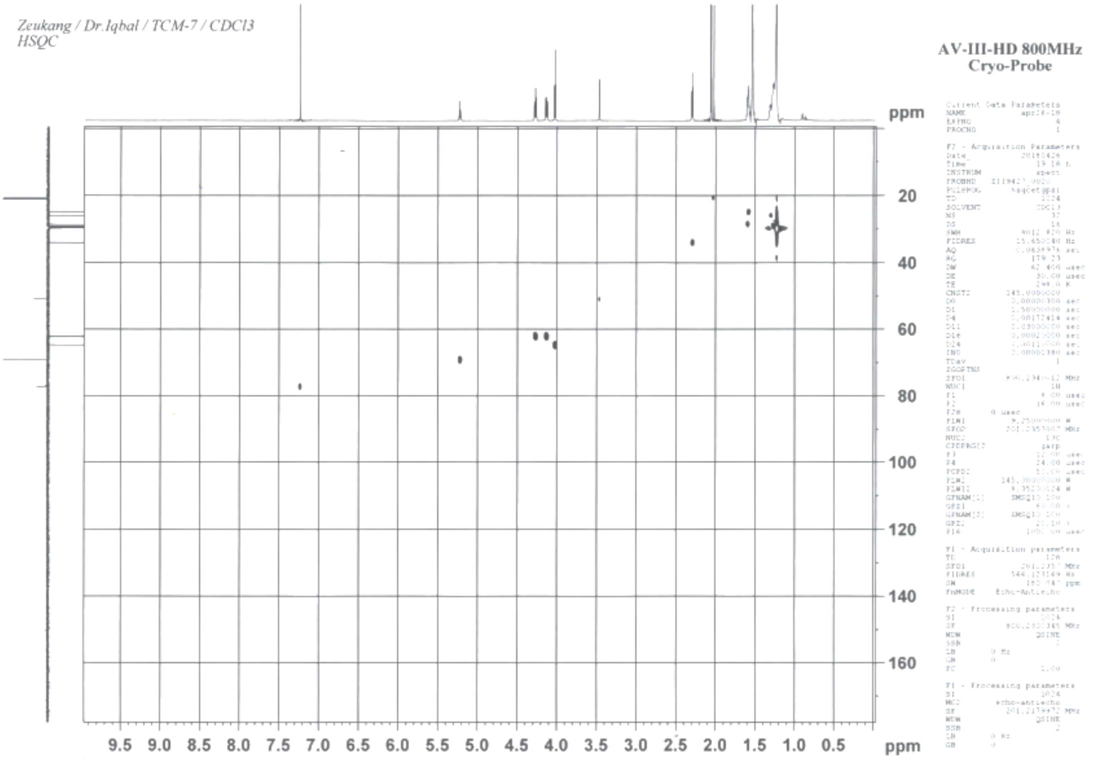

Figure S13. HSQC (^{1}H: 500 MHz, ^{13}C: 200 MHz, $CDCl_3$) spectrum of Cordicerol A (**14**)

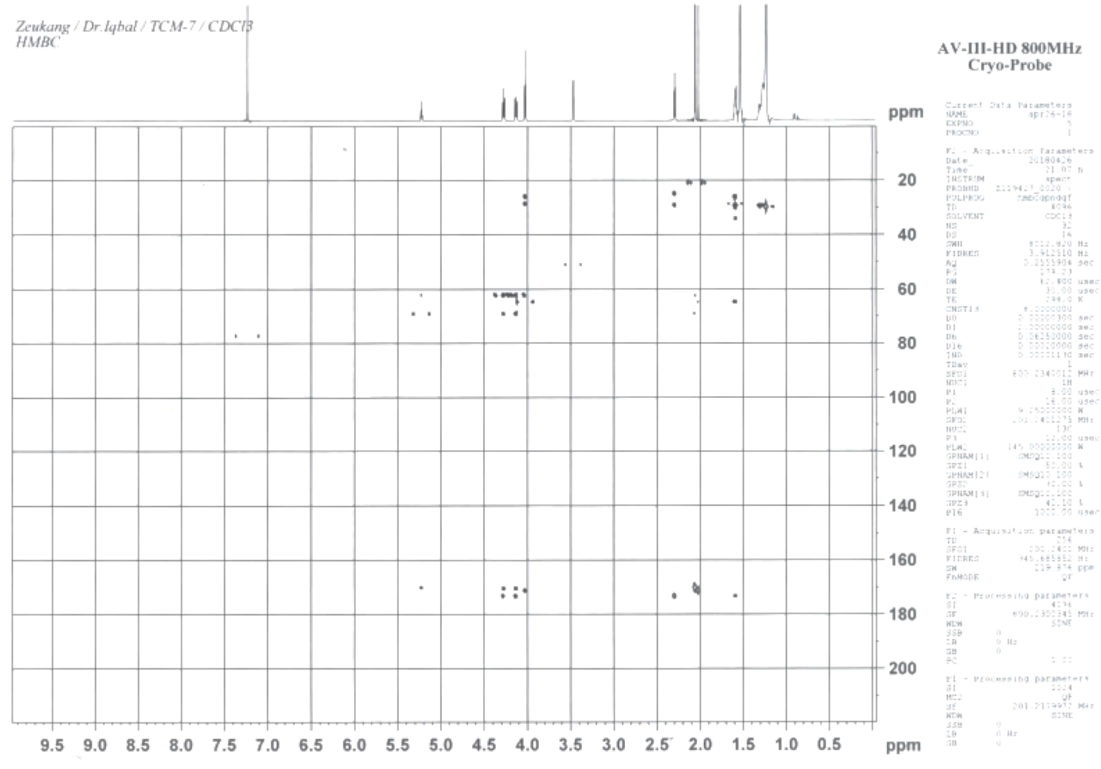

Figure S14. HMBC (^{1}H: 500 MHz, ^{13}C: 200 MHz, $CDCl_3$) spectrum of Cordicerol A (**14**)

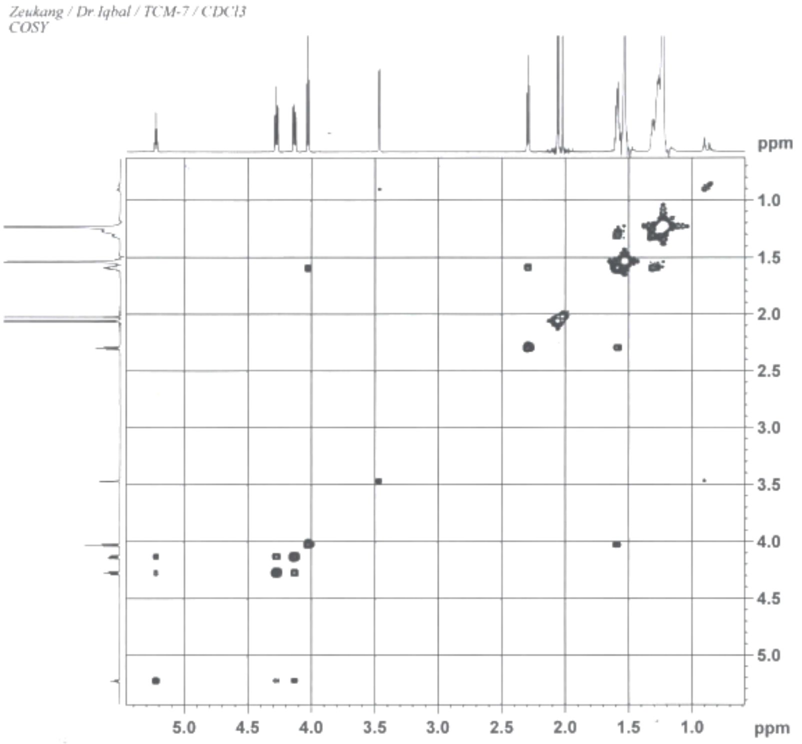

Figure S15. COSY (^{1}H: 500 MHz, $CDCl_3$) spectrum of Cordicerol A (**14**)

Compound 15 (Cordicerol B)

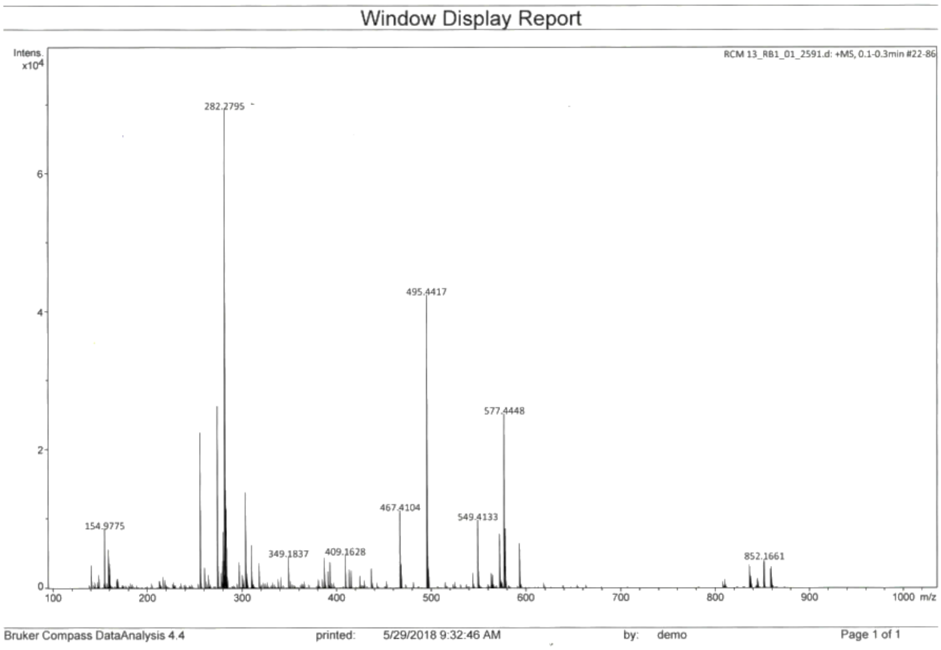

Figure S16. HR-ESI-MS of Cordicerol B (**15**)

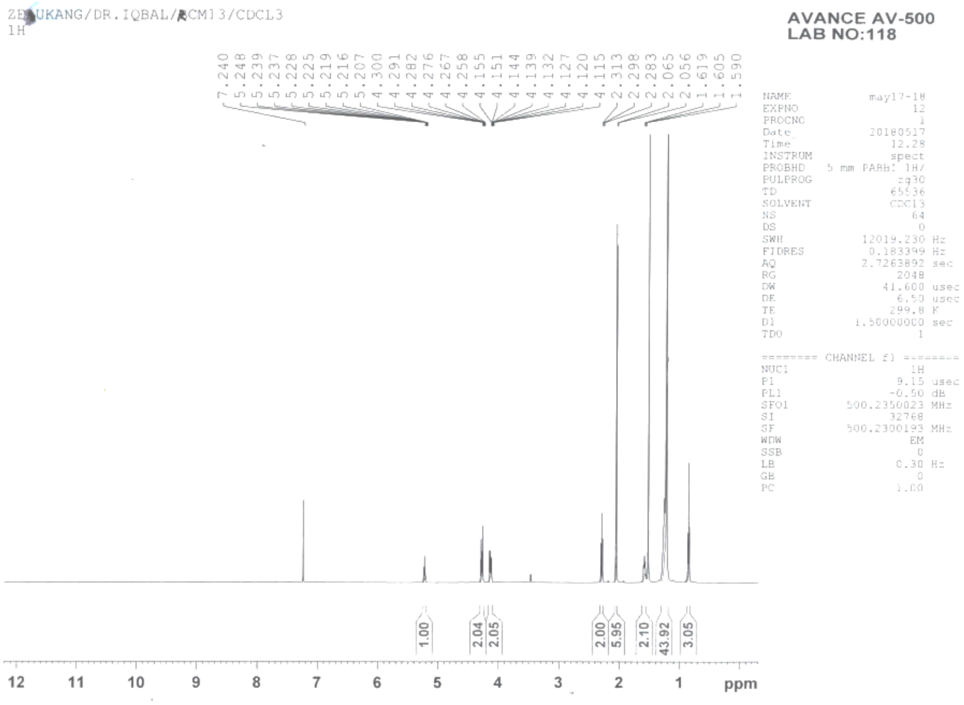

Figure S17. ^{1}H NMR (500 MHz, $CDCl_3$) spectrum of Cordicerol B (**15**)

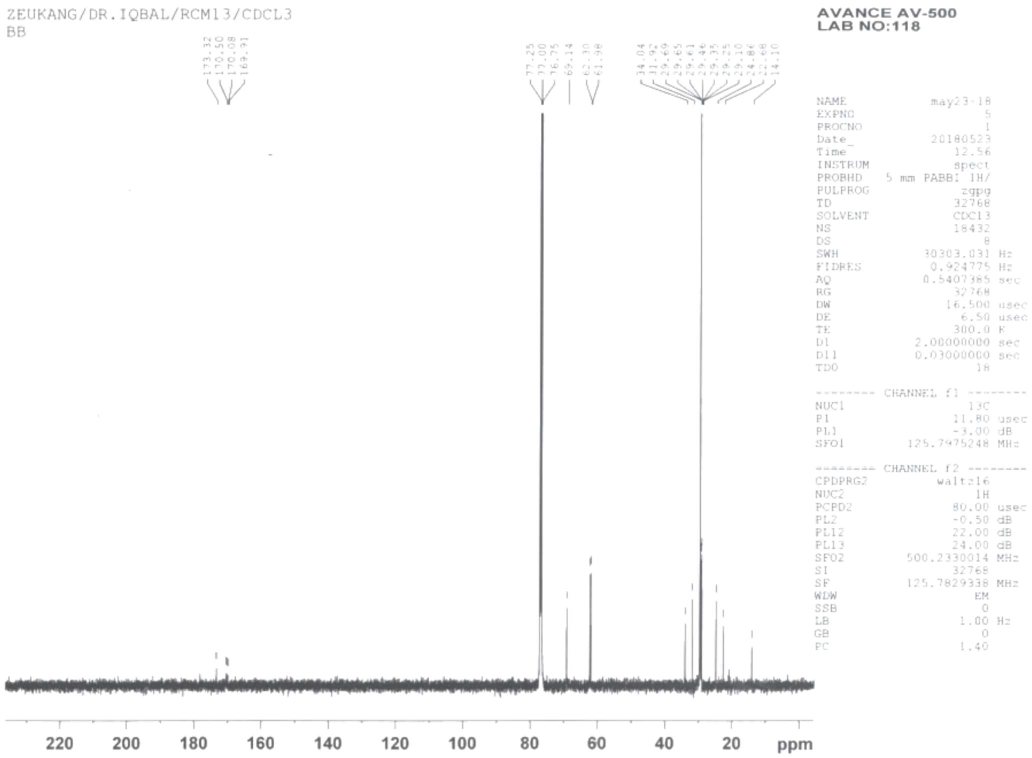

Figure S18. ^{13}C NMR (125 MHz, $CDCl_3$) spectrum of Cordicerol B (**15**)

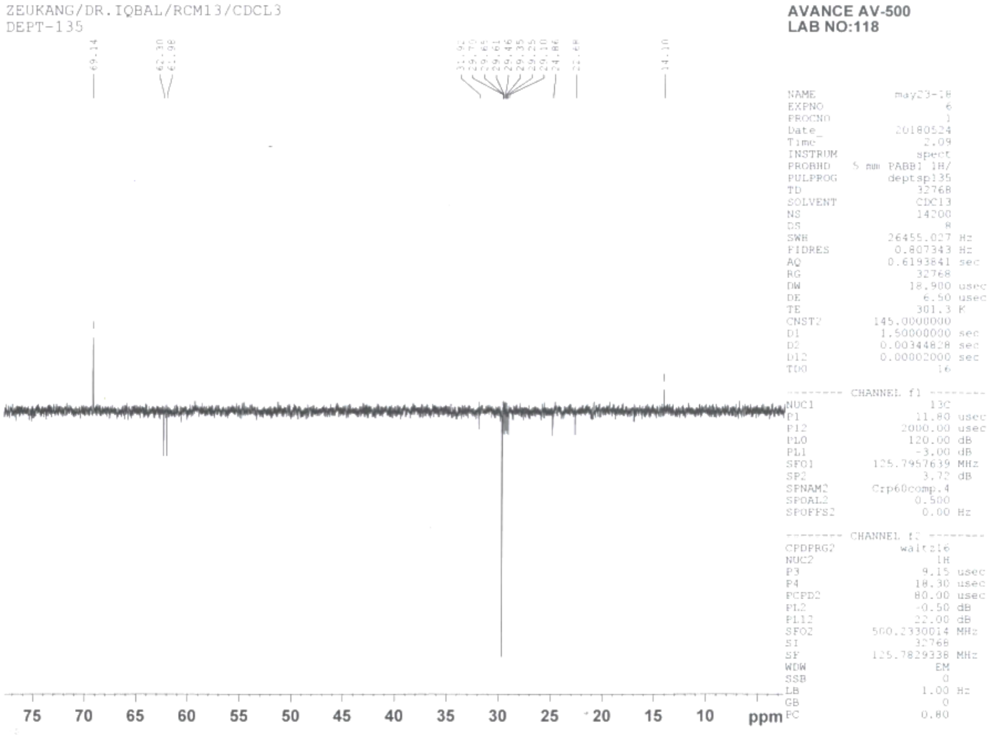

Figure S19. DEPT 135 (125 MHz, $CDCl_3$) spectrum of Cordicerol B (**15**)

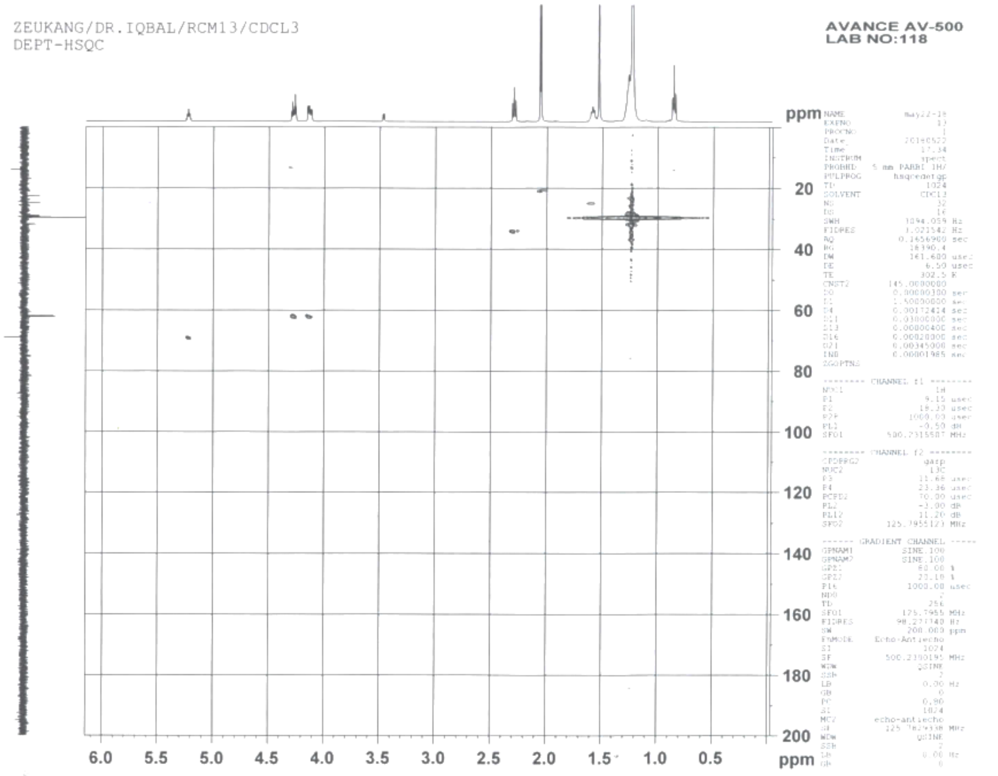

Figure S20. HSQC (^1H: 500 MHz, ^{13}C: 125 MHz, $CDCl_3$) spectrum of Cordicerol B (**15**)

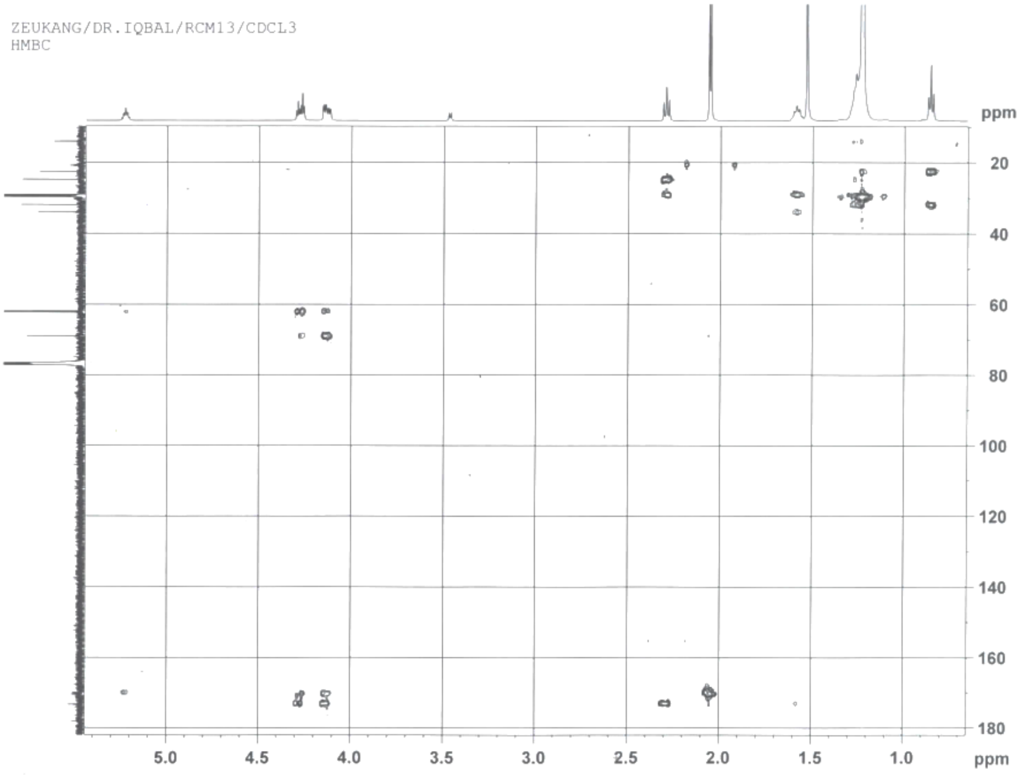

Figure S21. HMBC (^{1}H: 500 MHz, ^{13}C: 125 MHz, $CDCl_3$) spectrum of Cordicerol B (**15**)

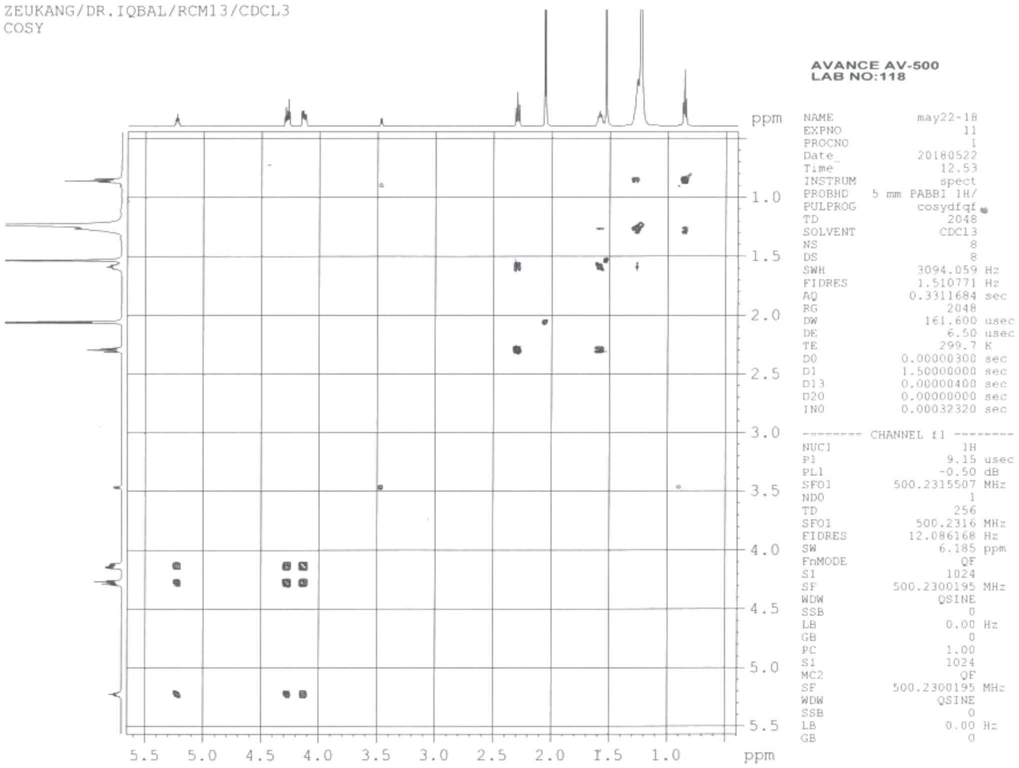

Figure S22. COSY (^{1}H: 500 MHz, $CDCl_3$) spectrum of Cordicerol B (**15**)

Compound 2 (Cyclooctasulfur)

Table S1. Crystal data and structure refinement for cyclooctasulfur (**2**).

	**2**
Formula	S8
Formula weight	256.48
T (K)	299(2)
λ (Å)	1.54178
Crystal system	Orthorhombic
Space group	Fddd
Unit cell dimensions	
a (Å)	10.4709(7)
b (Å)	12.8709(8)
c (Å)	24.484(2)
λ (°)	90
β (°)	90
γ (°)	90
Z	16
D_{calc} (Mg/m^3)	2.065
Absorption coefficient (mm^{-1})	19.279
F(000)	2048
Crystal size (mm)	0.16 x 0.07 x 0.06
θ (°)	5.74-66.64
Limiting indices	$-12 \leq h \leq 12$
	$-15 \leq k \leq 15$
	$-29 \leq l \leq 28$
Reflection collected/unique (R_{int})	10593/735 (0.0520)
Completeness to θ = 66.64	99.9%
Maximum and minimum transmission	0.3909 and 0.1484
Refinement method	Full-matrix least-squares on F^2
Data/restraints/parameters	735/0/8
Goodness-of-fit (GOF) on F^2	1.038
Final R indices [I>2σ(I)]	R1 = 0.0178
	wR2 = 0.0449
R indices (all data)	R1 = 0.0194
	wR2 = 0.0457
Largest diff. peak and hole (eÅ^{-3})	0.255 and -0.239

Table S2. Atomic coordinates (Å x 10^4) and equivalent isotropic displacement parameters (Å^2 x 10^3) for cyclooctasulfur (**2**).

Atoms	x	y	z	U(eq)
S(2)	428(1)	4798(1)	2459(1)	39(1)
S(4)	359(1)	6577(1)	3705(1)	37(1)
S(1)	-1058(1)	4528(1)	2986(1)	38(1)
S(3)	-341(1)	5303(1)	1738(1)	40(1)

S: Atoms sulfur
x, y and z : atomic coordinates
U(eq) is defined as one third of the trace of the orthogonalized Uij tensor.

Table S3. Bond lengths [Å] and angles [°] for cyclooctasulfur (**2**).

Bond lengths [Å]	angles [°]
S(2)-S(3)	2.0461(6)
S(2)-S(1)	2.0502(6)
S(4)-S(4)#1	2.0466(8)
S(4)-S(3)#2	2.0477(6)
S(1)-S(1)#3	2.0422(8)
S(3)-S(4)#4	2.0477(6)
S(3)-S(2)-S(1)	107.34(3)
S(4)#1-S(4)-S(3)#2	108.99(2)
S(1)#3-S(1)-S(2)	108.40(3)
S(2)-S(3)-S(4)#4	107.99(2)

Symmetry transformations used to generate equivalent atoms:
#1 -x+1/4,-y+5/4,z #2 -x,y+1/4,z+1/4
#3 -x-1/4,-y+3/4,z #4 -x,y-1/4,z-1/4

Table S4. Torsion angles [°] for cyclooctasulfur (**2**).

S(3)-S(2)-S(1)-S(1)#3	98.88(3)
S(1)-S(2)-S(3)-S(4)#4	-100.84(3)
